# Dual Anticancer and Antibacterial Applications of Active Particle Aggregates Containing Metal Phthalocyanines in Dark

**DOI:** 10.3390/ijms27167322

**Published:** 2026-08-16

**Authors:** Laila Jaragh-Alhdadad, Moayyad Alsawah, Rasheed Ahmad

**Affiliations:** 1 Department of Chemistry, College of Science, Kuwait University, Sabah Al Salem University City, Shadadiya, Kuwait City 13060, Kuwait; 2 Immunology & Microbiology Department, Dasman Diabetic Institute, Dasman, Kuwait City 15462, Kuwait

**Keywords:** metal phthalocyanine, particle aggregates, drug delivery, chitosan, PEG, LDL, anticancer assessment, antibacterial activity

## Abstract

Cancer is still a significant worldwide health concern, and the side effects of traditional chemotherapy emphasize the need for safer and more potent treatment methods. The structural diversity and specific biological action of bioorganic molecules make them essential components of many anticancer medications. Therefore, the anticancer and antibacterial activity of metal phthalocyanine agents were designed, synthesized, thoroughly characterized, and assessed in this study. These agents were loaded in both natural and artificial nanoparticles, such as chitosan (Chi), polyethylene glycol (PEG), and low-density lipoprotein (LDL), to improve therapeutic efficacy. Morphological and physicochemical properties were assessed using scanning electron microscopy (SEM), dynamic light scattering (DLS), energy-dispersive X-ray (EDX), and thermal gravimetric analysis (TGA). Complexes formed were increased in particle size and improved biological efficacy. Both free and metal phthalocyanine complexes were tested against breast (MDA-MB-453) and liver cancer (HEPG2) cell lines and *Escherichia coli* (*E. coli)* bacteria, with metal phthalocyanine complex formulations showing superior activity. Notably, gallium phthalocyanine (II) showed the strongest anticancer and antibacterial properties, indicating its potential as a promising future treatment option without light activation.

## 1. Introduction

Cancer and bacterial infections remain major health challenges due to their high mortality rates, necessitating extensive scientific investigation [1,2,3]. In recent years, a variety of organic compounds, including phthalocyanine [4], pyrazole [1], hydrazine [5,6], thiazole [7,8], triazole [3], and pyrimidine derivatives [9,10], have been investigated for their potential as anticancer and antibacterial agents [3,11]. These compounds exhibit diverse biological activities, including antibacterial, anticancer, antimalarial, anti-Alzheimer’s, and antiviral properties [12,13,14]. Current research is directed toward optimizing their molecular structures, solubility, and targeting capabilities to improve therapeutic efficacy and selectivity while reducing undesirable side effects [15,16].

Phthalocyanine is a synthetic macrocyclic molecule that shares structural similarities with the porphyrins found in heme and chlorophyll [4]. It has a central cavity that allows π–π interactions with metal ions, resulting in the creation of metal phthalocyanines [17]. It is a big aromatic molecule with great chemical and thermal stability [18]. The anticancer and antibacterial characteristics of phthalocyanines, especially metal derivatives like Zn, Al, Ga, Mg, and Cu complexes, have been extensively researched [18,19,20]. They function as photosensitizers in photodynamic treatment, causing cancer cells to die by producing reactive oxygen species when exposed to light ≈ 670–690 nm [4]. While reducing toxicity, structural alterations and nanoparticle encapsulation improve their tumor selectivity, cellular uptake, and therapeutic efficacy [4,21]. Similar to this, phthalocyanines have antibacterial activity against both Gram-positive and Gram-negative bacteria, through inducing membrane and DNA damage through reactive oxygen species (ROS) [22]. Their promise as multipurpose therapeutic agents is highlighted by nanoparticle encapsulation systems, which further enhance their solubility, stability, and cell penetration [23,24,25].

Drugs can be encapsulated in liposomes, nanoparticles [26,27], micelles, polymers, or hydrogels for active delivery [28]. Drug encapsulation is a vital strategy for targeted drug delivery, providing a protective shell or matrix around therapeutic compounds [9,24,28]. This approach enhances drug stability, enables controlled release, improves bioavailability, and reduces side effects, resulting in safer and more effective therapies [3,29,30]. Encapsulation and complex formation also protect sensitive bioactive substances from harsh environmental conditions, such as pH fluctuations, high temperatures, and enzymatic degradation [31,32]. Compared to conventional immediate-release formulations, it allows site-specific delivery, minimizes exposure to healthy tissues, and improves patient compliance while extending drug shelf life [28,33]. Natural low-density lipoprotein (LDL) exploits overexpressed LDL receptors on cancer cells for targeted uptake via receptor-mediated endocytosis [9,24,34]. Second, polyethylene glycol (PEG) synthetic nanoparticles improve circulation time, stability, and therapeutic efficacy, with several formulations already in clinical use passively entering the cells [35,36]. Third, chitosan, a natural polysaccharide, provides biocompatible, targeted nanoparticle passive delivery [37]. Together, these carriers enhance solubility, stability, cellular uptake, controlled release, and reduce toxicity [3,38,39]. In depth, encapsulating metal phthalocyanines in LDL, PEG, and chitosan nanoparticles enhances their anticancer and antibacterial activity by improving stability, cellular uptake, and passive delivery, enabling sustained nanozyme-mediated cytotoxic effects even in the absence of light [3,40,41,42].

Nanozymes are enzyme-mimicking nanomaterials that catalyze reactions cost-effectively and are widely used in diagnostics [43] and therapy, including anticancer and antibacterial applications [44,45]. Metal phthalocyanines, such as gallium and magnesium, exhibit nanozyme-like, ROS generation that induces cancer cell death, with some activity occurring even without light [46]. Recently Papadopoulos et al. showed that Ga(III) complexes are able to induce immunogenic cell death [47]. Similarly, other metal-based nanozymes (e.g., Au, Ag, Fe_3_O_4_, Cu, ZnO) facilitate ROS-mediated killing of tumor cells and bacteria [45,48,49]. Therefore, phthalocyanines encapsulated in nanoparticles demonstrate both direct and passive anticancer and antibacterial activity, even in the absence of light, due to their intrinsic chemical, catalytic, and nanozyme properties [49,50]. Iron, cobalt, copper, zinc, and manganese phthalocyanines generate ROS that cause DNA damage, mitochondrial dysfunction, and apoptosis in various cancer cells, while disrupting bacterial membranes and inhibiting enzymatic activity in *E. coli*, *S. aureus*, and *Pseudomonas aeruginosa* [51,52,53]. Encapsulation further enhances stability, cellular uptake, and sustained cytotoxicity [3], making metal phthalocyanine nanoparticles a promising platform for light-independent therapeutic applications [25].

**Figure 1:**
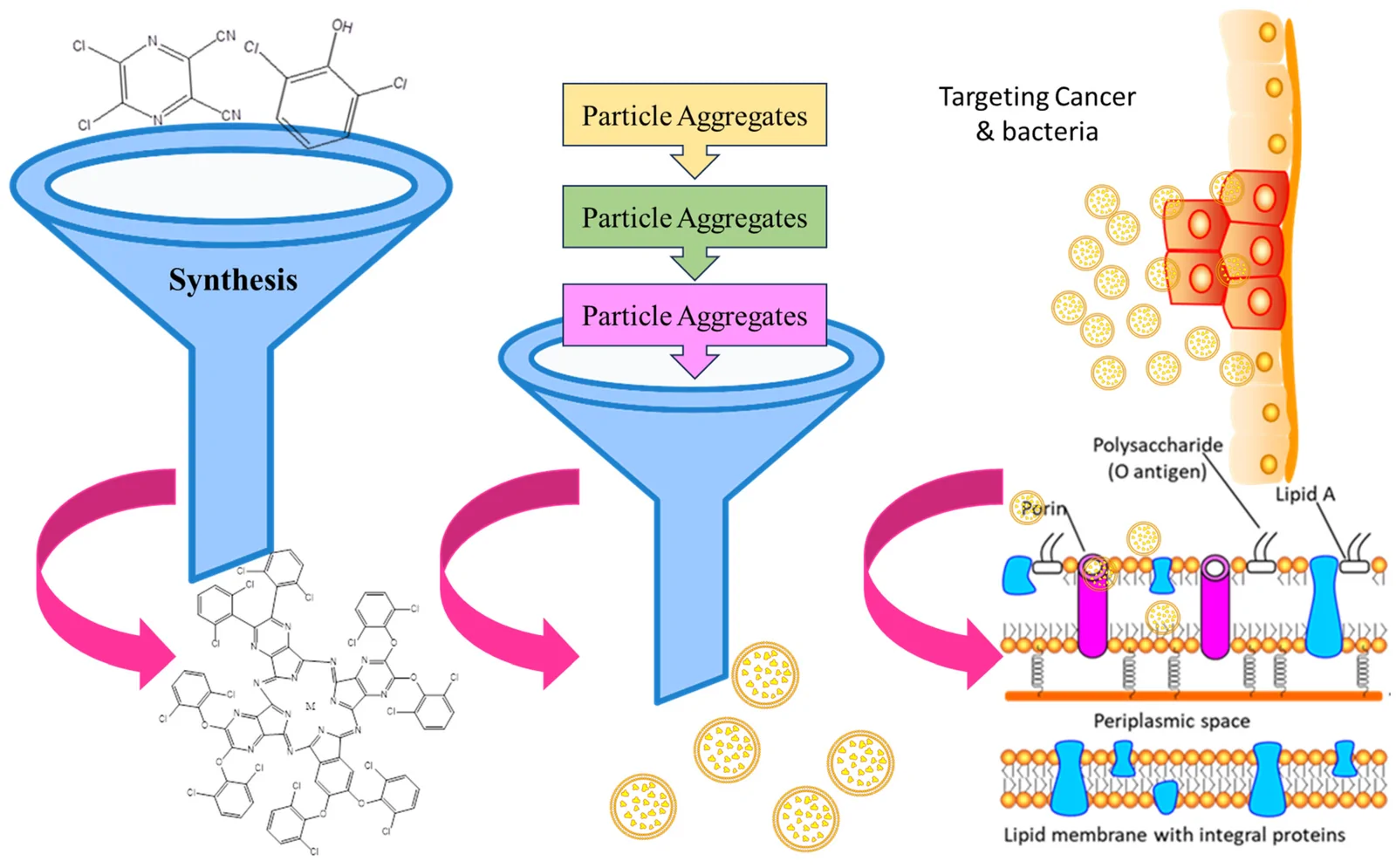
Metal phthalocyanines were synthesized and comprehensively characterized, followed by encapsulation and evaluation of their anticancer and antibacterial activities against cancer cell lines and *E. coli*.

In this project, we synthesized and fully characterized metal phthalocyanines mimicking the nanozyme activity using FAB-MS, NMR, UV–Vis, SEM, DLS, EDX, and TGA. After loading of the metal phthalocyanine into Chi, PEG, and LDL particles, the biological activities of these agents were evaluated against cancer cell lines (MDA-MB-453 and HEPG2) as well as *Escherichia coli* (*E. coli*) as a surrogate bacterial model, as represented in Figure 1.

## 2. Results

Following synthesis, comprehensive physicochemical characterization and biological evaluations of the metals phthalocyanine agent were performed to demonstrate the ability of the metal phthalocyanine to enter cancer and bacterial cells and exert activity in the absence of light activation.

### 2.1. Electronic Absorption Spectral Analysis

**Figure 2:**
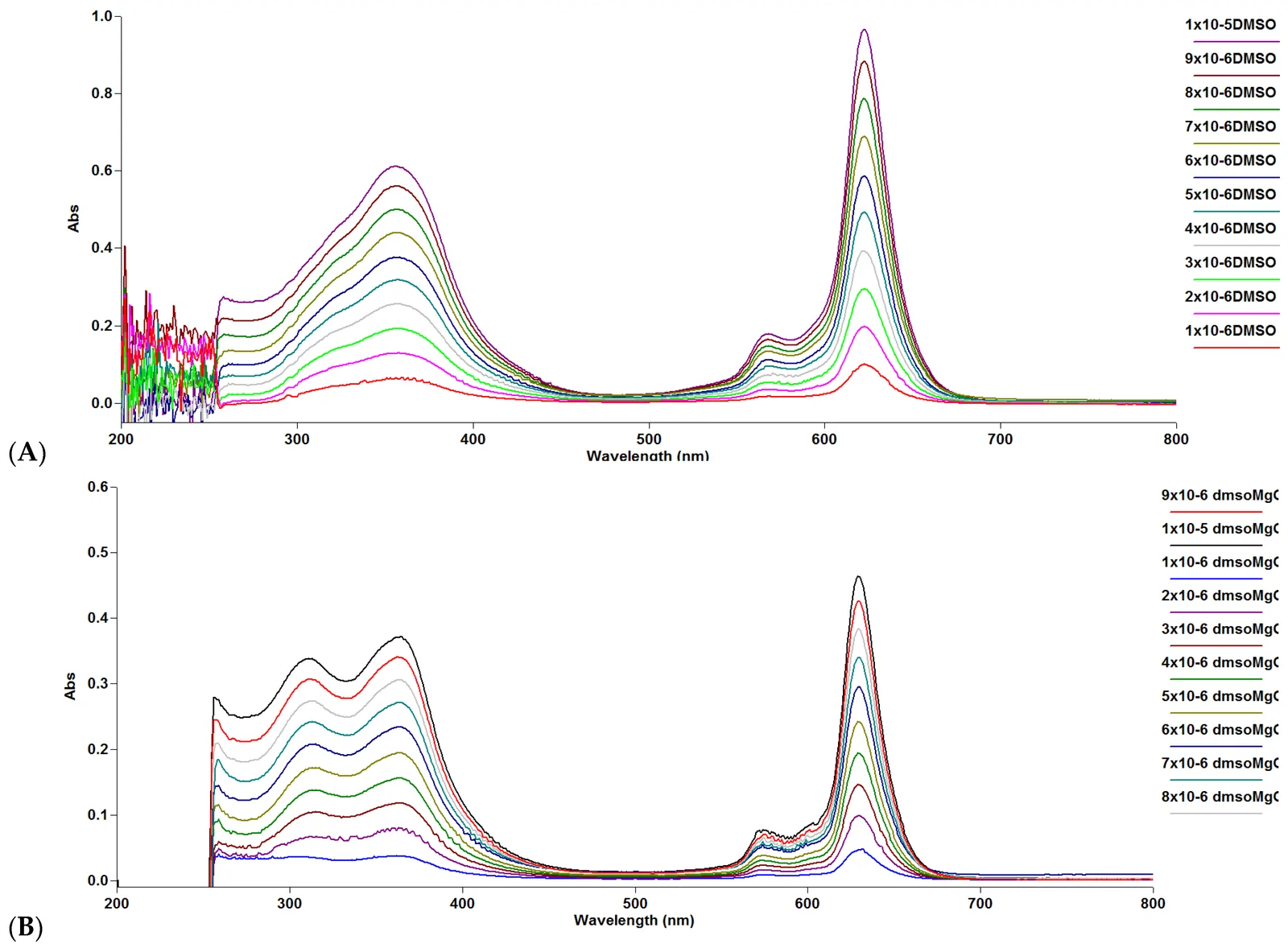
(**A**) Cu phthalocyanine and (**B**) Mg phthalocyanine showed consistent behavior across all tested doses, including a tenfold increase in concentration.

The UV–Vis spectra of all phthalocyanine agents exhibited distinct absorption peaks at around 350 nm and 650 nm [54], as summarized in Table 1. The absorption peaks of Cu phthalocyanine were at 358 and 622 nm, while Ga phthalocyanine showed absorption peaks at 315, 366 nm and 596–627 nm. Mg phthalocyanine absorption peaks were at 315–396, and 629 nm. In depth, the absorption peak near 330–370 nm is a B-band and originating from π → π* electronic transitions within the conjugated phthalocyanine macrocycle. Its presence confirms the integrity and aromaticity of the phthalocyanine ring system. On the other hand, the strong absorption band observed in the 600–700 nm region is known as the Q-band. This band arises from π → π* transitions involving the highest occupied molecular orbital (HOMO) and lowest unoccupied molecular orbital (LUMO) of the macrocycle [55,56]. Also, the exact position and intensity of the Q-band are influenced by the central metal ion, and this band is important because it lies in the visible to near-infrared region, making phthalocyanines suitable for applications in photodynamic therapy, photovoltaics, and sensors [57,58]. In sum, these bands confirmed the formation and the structural integrity of the metal phthalocyanine complexes. The structures were uniform even when increasing the concentrations ten times, as shown in Figure 2A,B. The spectra showed that phthalocyanine agents are in the monomeric form because even at high concentrations the data did not show broadening of the Q-band, reduction in the peak sharpness, or shift in the wavelength. Also based on the Beer–Lambert Law, the absorbance increases proportionally with the concentration [59].

### 2.2. Evaluation of Thermal Stability Using TGA

The purpose of thermal gravimetric analysis (TGA) is to compare agents’ temperatures to 37 °C (normal body temperature), and it tells us whether the compound remains intact in the human body [60]. If there is weight loss close to or below 37 °C, the compound may be unstable under physiological conditions, which could reduce efficacy or cause premature degradation. The TGA data demonstrated distinct thermal degradation profiles for the synthesized metal phthalocyanines. Cu phthalocyanine exhibited the onset of thermal degradation at approximately 111 °C, whereas Mg and Ga phthalocyanines showed higher degradation temperatures, initiating at around 125 °C. The elevated decomposition temperatures of Mg and Ga phthalocyanines indicate superior thermal stability compared to the Cu analogue. Importantly, all compounds remained thermally stable well above 100 °C, which is significantly higher than physiological temperature (37 °C). This substantial thermal margin confirms that these phthalocyanine-based anticancer agents would retain their structural integrity under biological conditions, supporting their suitability for use as chemotherapy candidates (Table 2).

This wide thermal safety margin strongly suggests that the phthalocyanines will remain structurally intact under in vivo conditions, including during systemic circulation, cellular uptake, and intracellular localization [4]. Furthermore, such thermal stability is critical for maintaining chemical integrity during formulation processes such as encapsulation, sterilization, and storage [61]. Also, it reduces the likelihood of premature degradation or loss of therapeutic efficacy in biological environments. Collectively, these findings support the suitability of the synthesized metal phthalocyanines as stable anticancer candidates for in vivo chemotherapy applications [4]. In sum, TGA analysis revealed that metal phthalocyanines exhibit enhanced thermal stability compared to body temperature. Minor weight loss below 150 °C corresponds to the removal of adsorbed moisture, while a broad stable region extending up to ~350 °C indicates strong structural integrity. The main decomposition step at higher temperatures is associated with macrocyclic ring degradation. The higher decomposition onset temperatures and increased residual mass of the metal phthalocyanines confirm that metal insertion significantly improves thermal robustness through strong metal–nitrogen coordination [62].

### 2.3. EDX Spectroscopic Characterization of the Agents

The energy-dispersive X-ray spectroscopy (EDX) identified and confirmed the presence of the elements in the sample in the analyzed area qualitatively and quantitatively. The samples were placed on double-face carbon tape and precoated with gold to enhance conductivity and signal quality. A comparison of the three phthalocyanine samples revealed that carbon was the dominant element in all cases, reflecting the aromatic and highly conjugated nature of the phthalocyanine macrocycle. Gallium phthalocyanine exhibited the highest carbon atomic percentage (88.13 at.%), followed by copper phthalocyanine (82.80 at.%) and magnesium phthalocyanine (69.44 at.%). Magnesium phthalocyanine was distinguished by a substantial nitrogen content (14.48 at.%), consistent with the nitrogen-rich structure of the phthalocyanine ring. The central metal atoms were detected in all samples, with copper showing the highest atomic percentage (1.21 at.%), followed by gallium (0.20 at.%) and magnesium (0.15 at.%). Overall, the elemental compositions are consistent with the expected characteristics of metal phthalocyanine materials, where a carbon-rich macrocyclic framework surrounds a centrally coordinated metal ion, as shown in Table 3.

### 2.4. SEM Microscopy Data Analysis of the Metals Phthalocyanine Agents

The micrographs of Cu, Mg and Ga phthalocyanine showed aggregates with rough and porous surfaces, as shown in Table 4. The data revealed that all agents were particle aggregates. Cu and Mg phthalocyanine morphologies were similar sharp and rough surfaces while Ga phthalocyanine showed sheet-like structures with the highest surface porosity. This porosity of the agents enables them to enhance the biological activity [63]. Porous materials are important in cancer therapy, as they can improve drug loading, facilitate tumor targeting, enable controlled release, and promote cellular uptake through both active and passive endocytosis, while also contributing directly to cancer cell killing through multiple mechanisms [64,65,66].

### 2.5. Metal Phthalocyanine Characterization

Dynamic light scattering (Zeta sizer) analysis revealed that the synthesized agents exhibited varied morphologies capable of disrupting microtubule stability [3,66,67], with smaller forms such as clusters, sheet-like assemblies, and particles with sharp-edged or porous surfaces which have been reported to interact and interfere with cellular microtubules, potentially disrupting the stability of α- and β-tubulin subunits and thereby inhibiting proper mitotic spindle formation and preventing cell division [67,68,69]. Collectively, these physicochemical characteristics may enhance cellular uptake and biological efficacy, supporting the observed anticancer and antibacterial activities of the prepared particle aggregates.

### 2.6. Nanoparticle Size Distribution Analysis Before and After Complex Formation

Dynamic light scattering (DLS) analysis was performed to evaluate the hydrodynamic particle size, polydispersity index (PDI), and intensity distribution of the free phthalocyanines and their complexes with low-density lipoprotein (LDL), polyethylene glycol (PEG), and chitosan nanoparticles. The native carriers exhibited average particle sizes of 78.10 ± 3.57 nm for LDL, 147.5 ± 9.75 nm for PEG, and 298.5 ± 28.76 nm for Chi. The size distribution data are summarized in Table 5. The results revealed that all agents became bigger after Chi, PEG, and LDL aggregate formation [3,34,35,36,37,38]. In depth, all agents loaded with LDL became bigger than the free LDL [9,24]. This trend is the same with PEG and chitosan encapsulation compared to the free PEG and chitosan as controls. Following complex formation, the particle sizes increased in most formulations, indicating successful association of the phthalocyanines with the respective nanocarriers. Cu phthalocyanine increased from 258.1 ± 64.74 nm to 324.5 ± 22.59 nm with LDL, 269.7 ± 28.42 nm with PEG, and 358.1 ± 24.71 nm with chitosan. Similarly, Mg phthalocyanine increased from 259.2 ± 66.86 nm to 329.6 ± 20.60 nm with LDL, while particle sizes of 263.4 ± 36.99 nm and 295.3 ± 3.82 nm were observed after complexation with PEG and chitosan, respectively. Among all formulations, Ga phthalocyanine exhibited the greatest increase in particle size, increasing from 159.5 ± 38.11 nm to 520.9 ± 95.74 nm with LDL, 597.7 ± 94.30 nm with PEG, and 450.4 ± 21.24 nm with chitosan, suggesting stronger interactions with the carrier matrices and a greater tendency toward nanoparticle aggregation.

The poly dispersive index (PDI) values revealed differences in particle size uniformity among the formulations. Native LDL exhibited a moderate PDI of 0.382, whereas free PEG and chitosan showed broader size distributions with PDIs of 1.000. Following complex formation, the PDI values ranged from 0.339 to 0.946, indicating varying degrees of particle homogeneity. The Ga-LDL formulation exhibited the lowest PDI (0.339), suggesting a comparatively more uniform particle population despite its larger particle size, while Cu-chitosan (0.920), Mg-LDL (0.932), and Ga-chitosan (0.946) displayed broader size distributions, indicative of greater heterogeneity and possible aggregation. The intensity-weighted distributions demonstrated that most formulations were dominated by a single particle population (100% intensity), confirming the formation of the desired particle complexes. However, Cu-PEG (71% intensity) and Ga-PEG (55% intensity) exhibited additional particle populations, indicating the coexistence of particles with different sizes or the presence of aggregates. As DLS intensity distributions are strongly influenced by larger particles, these lower-intensity percentages likely reflect minor aggregated populations rather than a reduction in particle formation. Overall, the DLS results confirm successful complexation of the phthalocyanines with LDL, PEG, and chitosan carriers, with gallium phthalocyanine producing the largest hydrodynamic particle sizes and the greatest influence on particle size distribution.

### 2.7. Quantitative Analysis of MTT Assay Results

The cytotoxic effects of Cu, Mg, and Ga phthalocyanines and their particle-based formulations were evaluated against the MDA-MB-453 breast cancer cell line, with HEK293 normal human cells serving as a non-cancerous control. All the culture results are summarized in Table 6. The data revealed that Cu and Mg phthalocyanine did not show potent cell growth inhibition when treated with breast cancer cell line at 25 µM, while Ga phthalocyanine showed potent cancer cell growth at 3.965 ± 0.097 µM IC_50_ value against breast cancer cells. The results demonstrated that particle formulation significantly enhanced the anticancer activity of all tested phthalocyanines, as evidenced by substantial reductions in IC_50_ values compared with the free compounds. As lower IC_50_ values indicate greater cytotoxic potency, the LDL-loaded formulations consistently exhibited the strongest anticancer effects among the tested delivery systems [3]. First, loading of the agents with LDL showed that Ga > Mg > Cu. Second, loading of the agents with PEG showed that Ga > Cu > Mg. Third, loading with chitosan showed the same trend as LDL Ga > Mg > Cu. For Cu phthalocyanine, the free compound displayed relatively weak activity against MDA-MB-453 cells, with an IC_50_ value exceeding 20 µM. However, encapsulation within LDL nanoparticles markedly improved its cytotoxic efficacy, reducing the IC_50_ to 1.478 ± 0.144 µM. PEG loading also enhanced activity, yielding an IC_50_ of 2.86 ± 0.113 µM, whereas chitosan loading did not produce a comparable improvement, with the IC_50_ remaining above 20 µM. These findings indicate that LDL-mediated delivery substantially increased the intracellular effectiveness of Cu phthalocyanine, likely through enhanced cellular uptake by breast cancer cells. A similar trend was observed for Mg phthalocyanine. The free compound exhibited limited cytotoxicity, with an IC_50_ value exceeding 20 µM. Following LDL encapsulation, the IC_50_ decreased dramatically to 1.319 ± 0.151 µM, representing the most pronounced enhancement among the Mg-based formulations. PEG loading also improved activity, although to a lesser extent (IC_50_ = 5.402 ± 0.120 µM), while chitosan loading produced an intermediate effect with an IC_50_ of 8.597 ± 0.120 µM. These results demonstrate that particle-mediated delivery significantly potentiates the anticancer activity of Mg phthalocyanine, with LDL particles providing the greatest enhancement. Among all compounds investigated, Ga phthalocyanine exhibited the highest intrinsic anticancer activity. The free compound achieved an IC_50_ value of 3.965 ± 0.097 µM, which was considerably lower than those of free Cu and Mg phthalocyanines. Incorporation into LDL nanoparticles further enhanced its potency, producing the lowest IC_50_ value observed in the entire study (1.030 ± 0.112 µM). PEG and chitosan formulations also improved cytotoxicity, yielding IC_50_ values of 2.114 ± 0.143 µM and 3.207 ± 0.104 µM, respectively. The superior activity of Ga phthalocyanine suggests that the gallium center contributes favorably to biological interactions and cellular toxicity toward breast cancer cells, while LDL encapsulation maximizes its therapeutic efficacy.

The data revealed that LDL complex formation showed potent IC_50_ values when breast cancer cells were treated with all metal phthalocyanine agents followed by PEG encapsulation then chitosan nanoparticles. This enhanced efficacy may be attributed to the elevated expression of low-density lipoprotein receptors (LDLRs) on many breast cancer cells, including MDA-MB-453 cells, which facilitate receptor-mediated endocytosis and selective accumulation of LDL-associated therapeutic agents within malignant tissues. Consequently, LDL nanoparticles appear to provide a targeted delivery mechanism that enhances intracellular drug concentration and therapeutic response.

Evaluation of cytotoxicity toward HEK293 normal cells further demonstrated favorable selectivity for cancer cells. The free phthalocyanines exhibited substantially higher IC_50_ values against HEK293 cells, ranging from 29.36 ± 0.215 to 35.11 ± 0.142 µM, compared with their corresponding IC_50_ values in MDA-MB-453 cells. Ga phthalocyanine, despite being the most potent anticancer agent, maintained an IC_50_ of 29.36 ± 0.215 µM in HEK293 cells, indicating a wide therapeutic window. The approximate selectivity indices (HEK293 IC_50_/MDA-MB-453 IC_50_) for the free compounds were >1.75 for Cu phthalocyanine, >1.66 for Mg phthalocyanine, and 7.41 for Ga phthalocyanine, demonstrating particularly strong cancer cell selectivity for the gallium derivative.

Overall, the data identify Ga phthalocyanine as the most potent anticancer agent, while LDL nanoparticles represent the most effective drug delivery system. The LDL-loaded Ga phthalocyanine formulation exhibited the greatest cytotoxic activity against MDA-MB-453 cells, achieving an IC_50_ of 1.030 ± 0.112 µM, nearly fourfold lower than that of the free compound. These findings suggest that combining gallium phthalocyanine with LDL-mediated delivery may represent a promising strategy for targeted breast cancer therapy, offering enhanced anticancer efficacy while maintaining a favorable selectivity profile toward normal cells.

The anticancer activities of Cu, Mg, and Ga phthalocyanines and their corresponding particle formulations were evaluated against the HepG2 liver cancer cell line, while HEK293 cells were used as a normal cell control to assess selectivity. The results demonstrated that both the nature of the central metal ion and the choice of nanocarrier significantly influenced cytotoxic efficacy. Overall, LDL-based formulations consistently produced the lowest IC_50_ values, confirming their superior drug delivery performance, whereas Ga phthalocyanine emerged as the most potent anticancer agent among the investigated compounds. The substantial differences between the IC_50_ values obtained for HepG2 and HEK293 cells further indicate a favorable therapeutic profile toward malignant cells, the data summarized in Table 7. First, loading of the agents with LDL showed the same trend as the breast cancer treatment: Ga > Mg > Cu. Second, loading of the agents with PEG also showed that Ga > Cu > Mg. Third, loading with chitosan showed the same trend as LDL loading: Ga > Mg > Cu [70]. All complex formation had an agent to carrier ratio of 1:5. Cu phthalocyanine exhibited relatively weak intrinsic activity against HepG2 cells, with an IC_50_ value exceeding 20 µM when administered in its free form. However, encapsulation within nanocarriers markedly improved its cytotoxic performance. LDL-loaded Cu phthalocyanine demonstrated the greatest enhancement, reducing the IC_50_ to 2.108 ± 0.111 µM, while PEG and chitosan formulations yielded IC_50_ values of 3.450 ± 0.109 µM and 8.610 ± 0.111 µM, respectively. These findings indicate that particle-mediated delivery substantially increases the biological effectiveness of Cu phthalocyanine, with LDL particles producing approximately a tenfold improvement in activity relative to the free compound. The enhanced efficacy may be attributed to improved cellular internalization and more efficient intracellular drug accumulation. Mg phthalocyanine displayed considerably greater intrinsic cytotoxicity than Cu phthalocyanine, exhibiting an IC_50_ value of 3.616 ± 0.110 µM against HepG2 cells. Loading in LDL particles further enhanced its activity, resulting in an IC_50_ of 1.296 ± 0.111 µM. In contrast, PEG- and chitosan-based formulations produced IC_50_ values of 7.906 ± 0.136 µM and 8.483 ± 0.100 µM, respectively, both of which were less effective than the free compound. This observation suggests that while LDL particles significantly improve the delivery and potency of Mg phthalocyanine, PEG and chitosan matrices may reduce its bioavailability or alter its interaction with cancer cells. Nevertheless, the LDL-loaded Mg phthalocyanine formulation remained among the most effective treatments evaluated, demonstrating nearly a threefold increase in potency relative to the free drug. Ga phthalocyanine exhibited the strongest intrinsic anticancer activity against HepG2 cells, achieving an IC_50_ value of 1.401 ± 0.101 µM. This potency was further enhanced following LDL complex formation, producing the lowest IC_50_ value observed in the study (1.193 ± 0.102 µM). PEG loading maintained a high level of activity, with an IC_50_ value of 1.754 ± 0.103 µM, whereas chitosan loading resulted in a moderate reduction in efficacy (IC_50_ = 3.221 ± 0.130 µM). The consistently low IC_50_ values observed for all Ga phthalocyanine formulations indicate that the gallium-centered phthalocyanine possesses strong inherent cytotoxic properties and remains highly effective regardless of the delivery platform. The slight but measurable improvement achieved through LDL complex formation suggests that the compound already possesses excellent cellular accessibility, while LDL delivery provides an additional enhancement in therapeutic efficiency.

Comparison of the three delivery systems revealed a clear trend in anticancer performance. LDL particles consistently produced the lowest IC_50_ values for all phthalocyanines, confirming their superiority as drug carriers. PEG formulations generally showed intermediate effectiveness, whereas chitosan-based formulations produced the highest IC_50_ values in most cases. The superior performance of LDL particles may be related to receptor-mediated uptake mechanisms, as cancer cells often exhibit increased lipid requirements and elevated expression of LDL receptors to support rapid proliferation [39]. Consequently, LDL-associated phthalocyanines can be internalized more efficiently, resulting in higher intracellular drug concentrations and enhanced cytotoxic activity [71]. LDL entered both cancer cells through binding to the LDL receptor like a key and lock [3,72]. In sum, LDL nanoparticles were the most potent complex formation strategy that entered the cells and caused cancer cell death.

Assessment of cytotoxicity toward HEK293 cells demonstrated that all three phthalocyanines were substantially less toxic to normal cells than to HepG2 cancer cells. The IC_50_ values against HEK293 cells ranged from 24.01 ± 0.327 to 26.22 ± 0.429 µM, considerably higher than those recorded for HepG2 cells. Ga phthalocyanine exhibited an IC_50_ of 24.32 ± 0.485 µM in HEK293 cells compared with 1.401 ± 0.101 µM in HepG2 cells, corresponding to an approximate selectivity index of 17.4 (healthy cells/cancer cells). Similarly, Mg phthalocyanine demonstrated a selectivity index of approximately 7.3, while Cu phthalocyanine exhibited a selectivity index greater than 1.2 due to its lower intrinsic activity against HepG2 cells. These findings indicate that Ga phthalocyanine possesses the most favorable balance between potency and selectivity, producing strong cytotoxic effects in cancer cells while maintaining comparatively low toxicity toward healthy cells.

Overall, the results identify Ga phthalocyanine as the most potent anticancer agent against HepG2 liver cancer cells, owing to its exceptionally low IC_50_ values in both free and formulated forms. Furthermore, LDL particles were the most effective delivery system, consistently enhancing the cytotoxic activity of all tested phthalocyanines. The LDL-loaded Ga phthalocyanine formulation achieved the greatest anticancer efficacy, with an IC_50_ value of 1.193 ± 0.102 µM, while retaining a wide therapeutic margin relative to HEK293 cells. These findings highlight the potential of gallium phthalocyanine, particularly when delivered through LDL nanoparticles, as a promising candidate for targeted liver cancer therapy.

### 2.8. In Vitro Antibacterial Evaluation of Metal Phthalocyanines

The antibacterial activity of Cu, Mg, and Ga phthalocyanines, both in their free form and following encapsulation within LDL, PEG, and chitosan carriers, was evaluated against *Escherichia coli* using the agar diffusion method. The antibacterial experimental data is summarized in Table 8. The results demonstrated generally low antibacterial activity for all tested formulations, as evidenced by the absence of distinct inhibition zones in most treatments. Free Cu, Mg, and Ga phthalocyanines did not produce detectable inhibitory effects against *E. coli* at 10 µM, indicating that the agents alone were unable to effectively suppress bacterial growth under the experimental conditions employed. Similarly, LDL and PEG formulations failed to generate measurable inhibition zones at 10 µM, suggesting that these delivery systems did not significantly enhance the antibacterial efficacy of the phthalocyanines against this Gram-negative bacterium. In contrast, chitosan formulations exhibited detectable, although limited, antibacterial activity. Chitosan-loaded Cu phthalocyanine produced an inhibition zone of 1.8 mm, while chitosan-loaded Mg phthalocyanine generated a slightly smaller zone of 1.6 mm at 10 µM. The most pronounced antibacterial response was observed for chitosan-loaded Ga phthalocyanine, which produced an inhibition zone of 2.2 mm at 10 µM. Despite these measurable effects, the inhibition zones were described as weak and poorly defined, indicating only marginal suppression of bacterial growth. The absence of clear inhibition boundaries suggests that the antibacterial action was insufficient to produce strong bactericidal or bacteriostatic effects when using low concentration of the agents. The superior performance of the chitosan-based formulations relative to LDL and PEG carriers may be attributed to the intrinsic antimicrobial properties of chitosan. As a positively charged biopolymer, chitosan can interact electrostatically with the negatively charged bacterial cell envelope, increasing membrane permeability and disrupting normal cellular functions. This interaction may facilitate closer contact between the complex phthalocyanines and bacterial cells, thereby enhancing antibacterial activity. Nevertheless, the small inhibition zones observed indicate that the contribution of chitosan was limited and that the overall susceptibility of *E. coli* to these formulations remained low [70].

Among the tested compounds, Ga phthalocyanine exhibited the greatest antibacterial activity when delivered through chitosan nanoparticles, followed by Cu and Mg phthalocyanines. This trend may reflect differences in the physicochemical properties of the central metal ions, which can influence interactions with bacterial membranes and intracellular targets. However, the relatively small differences among the inhibition zones suggest that the type of carrier had a more pronounced influence on antibacterial performance than the specific phthalocyanine derivative itself. The limited antibacterial efficacy observed against *E. coli* is consistent with the well-documented resistance of Gram-negative bacteria to many antimicrobial agents. The outer membrane of *E. coli*, which contains lipopolysaccharides, acts as an effective permeability barrier that restricts the penetration of large molecules and aggregates-associated agents. Consequently, the phthalocyanine formulations were largely unable to reach intracellular targets at concentrations sufficient to produce substantial bacterial inhibition. Overall, the findings indicate that while chitosan encapsulation provided a modest enhancement in antibacterial activity, all tested formulations exhibited only weak inhibitory effects against *E. coli*, with chitosan-loaded Ga phthalocyanine representing the most active formulation among those evaluated.

To further evaluate the antibacterial potential of the synthesized metal phthalocyanines, *E. coli* cultures were exposed to higher treatment concentrations. At 50 µM, a substantial enhancement in antibacterial activity was observed for all tested formulations. The inhibition zone diameters for Cu, Mg, and Ga phthalocyanines ranged from 1.3 to 3.0 cm, 1.2 to 2.9 cm, and 1.3 to 3.0 cm, respectively, indicating a concentration-dependent increase in bacterial growth inhibition. Further increasing the concentration to 70 µM resulted in an even greater antibacterial response, with inhibition zone diameters expanding to 1.7–3.4 cm for Cu phthalocyanine, 1.7–3.2 cm for Mg phthalocyanine, and 2.0–3.5 cm for Ga phthalocyanine. Among the investigated compounds, Ga phthalocyanine exhibited the strongest antibacterial performance at the highest concentration tested.

The observed increase in inhibition zone diameter with increasing concentration demonstrates a clear dose-dependent antibacterial effect of the metal phthalocyanines against *E. coli*. Moreover, loading formulations consistently exhibited superior antibacterial activity compared with their free counterparts, highlighting the effectiveness of the carrier systems in enhancing the biological performance of these agents [30,32,34]. Improved dispersion, increased surface interaction with bacterial cells, and enhanced delivery of the active agents likely contributed to the observed enhancement in antibacterial efficacy. Collectively, these findings demonstrate that metal phthalocyanines, particularly when incorporated into carrier systems, possess significant antibacterial potential, and can produce pronounced and well-defined growth inhibition against *E. coli* at elevated concentrations (Table 9) [36,39].

## 3. Discussion

Metal phthalocyanines have traditionally been investigated as photosensitizers for photodynamic therapy because of their strong absorption in the visible and near-infrared regions and their ability to generate reactive oxygen species (ROS) upon light activation. However, increasing evidence suggests that selected metallophthalocyanines may also exert biological activity in the absence of external irradiation through redox-mediated processes, disruption of intracellular homeostasis, and metal-dependent biochemical interactions, additionally inhibiting iron-dependent pathways due to Fe^3+^ mimicry [45,49,73]. In the present study, we demonstrate that Cu-, Mg-, and particularly Ga-phthalocyanine formulations possess significant anticancer activity under dark conditions, and that nanoencapsulation within LDL, PEG, and chitosan carriers substantially enhances their biological performance [74,75,76,77]. These findings expand the therapeutic potential of phthalocyanine-based systems beyond conventional photodynamic applications and support their development as multifunctional nanotherapeutics.

The physicochemical characterization data provide important insights into the observed biological activities. UV–Vis spectroscopy confirmed preservation of the characteristic B and Q bands of the phthalocyanine macrocycle, indicating successful synthesis and structural integrity of the metal complexes [55]. Importantly, the absence of significant spectral broadening at elevated concentrations suggests limited aggregation and preservation of monomeric species, a feature often associated with improved biological performance and more predictable cellular interactions. Furthermore, TGA analysis demonstrated high thermal stability, with decomposition temperatures substantially exceeding physiological conditions, indicating that the complexes are likely to remain structurally intact during circulation, cellular uptake, and intracellular trafficking. Such stability is particularly important for therapeutic applications because premature degradation can compromise bioavailability and reduce therapeutic efficacy [3,60].

Further, the EDX spectroscopy qualitatively and quantitatively confirmed the agents’ components [3]. SEM and DLS analyses revealed particle aggregates possessing rough and porous surface morphologies. These structural characteristics are increasingly recognized as important determinants of nanomedicine performance Porous architectures provide greater surface area for biological interactions, facilitate adsorption to cellular membranes, and may enhance intracellular accumulation through endocytic pathways [9,24]. In addition, porosity can kill cancer cells via several mechanisms: either drug delivery, photothermal therapy or gene delivery. Also, porous compounds can target DNA, RNA, and induce apoptosis [78]. Moreover, studies have shown that porous particles can target *E. coli* bacteria through a combination of physiochemical surface modifications such as liposaccharides, positive selective binding, and responsive release mechanism such as acidic pH or enzyme sensitive linkers [79], and the advantages are controlled release, reduced resistance development, high loading capacity of the drug, and improved solubility and stability [80]. For example, mesoporous silica nanoparticles functionalized with cationic polymers and loaded with silver ions have been shown to selectively bind to *E. coli* cell walls and kill them via membrane disruption and ROS production [81]. Furthermore, nanoparticles offer significant advantages in cancer therapy and antibacterial applications due to their unique properties [26,27]. In cancer, they enable targeted drug delivery, improve solubility and stability, allow controlled release, and enhance cellular uptake, reducing systemic toxicity [3,28]. Also, in antibacterial studies, nanoparticles can disrupt bacterial membranes, induce oxidative stress, overcome antibiotic resistance, provide targeted delivery, and penetrate biofilms that are typically resistant to conventional antibiotics [82,83,84].

The marked increase in hydrodynamic diameter following incorporation into LDL, PEG, and chitosan carriers confirms successful formulation and suggests strong interactions between the phthalocyanines and carrier matrices. Notably, gallium II phthalocyanine produced the largest particle size increases after complex formation, indicating stronger carrier association and potentially improved cellular retention. Such characteristics may partially explain the superior biological activity observed for the gallium II phthalocyanine formulations.

Among the investigated compounds, gallium phthalocyanine consistently exhibited the highest anticancer activity against both MDA-MB-453 breast cancer and HepG2 liver cancer cells [85]. The superior efficacy of the gallium derivative may originate from several complementary mechanisms. Gallium shares chemical similarities with ferric iron (Fe^3+^) and can interfere with iron-dependent cellular processes that are essential for tumor proliferation. Rapidly growing cancer cells exhibit elevated iron requirements for DNA synthesis, mitochondrial function, and metabolic activity [86]. Consequently, intracellular gallium accumulation can disrupt iron metabolism, inhibit ribonucleotide reductase activity, induce oxidative stress, and, ultimately, trigger apoptosis. These mechanisms likely act synergistically with the intrinsic redox properties of the phthalocyanine scaffold, producing enhanced cytotoxic effects even in the absence of photoactivation. The exceptionally low IC_50_ values observed for gallium phthalocyanine therefore suggest that the metal center itself contributes significantly to biological activity rather than serving solely as a structural component of the macrocycle.

A particularly important finding of this study is the substantial enhancement in anticancer activity achieved through complex formation. Across both cancer models, LDL-based formulations consistently produced the lowest IC_50_ values, outperforming PEG and chitosan carriers [34,36,39]. This observation is consistent with the metabolic demands of malignant cells, which frequently overexpress LDL receptors to support increased cholesterol uptake required for membrane biosynthesis and rapid proliferation [9,24]. Exploiting this biological pathway enables receptor-mediated endocytosis and preferential intracellular delivery of LDL-associated therapeutics. The nearly fourfold improvement observed for LDL-loaded gallium II phthalocyanine in breast cancer cells and the similarly enhanced activity in HepG2 cells strongly support this targeting mechanism. These findings are particularly relevant because they demonstrate that naturally occurring lipoproteins can function as efficient and potentially biocompatible drug-delivery vehicles for metallo phthalocyanine-based therapeutics.

Although PEGylated formulations generally improved anticancer activity relative to the free compounds, their performance remained inferior to LDL-based systems. PEG is known to improve colloidal stability, reduce opsonization, and prolong systemic circulation; however, it lacks the active targeting capability provided by LDL-receptor-mediated uptake [39]. Consequently, the observed improvements are likely attributable primarily to enhanced solubility and cellular exposure rather than selective tumor targeting. Chitosan formulations produced variable responses depending on the metal center, suggesting that electrostatic interactions alone may be insufficient to maximize intracellular delivery of all phthalocyanine derivatives. These observations highlight the importance of matching carrier properties to both the physicochemical characteristics of the therapeutic agent and the biological features of the target cells.

An additional strength of the present work is the favorable selectivity observed toward cancer cells compared with HEK293 normal cells. Gallium II phthalocyanine displayed the highest selectivity indices among all tested agents, indicating that enhanced potency was not accompanied by proportional toxicity toward non-malignant cells. Such selectivity is a critical requirement for clinical translation because many conventional chemotherapeutics exhibit severe dose-limiting toxicities resulting from poor discrimination between healthy and malignant tissues. The combination of strong cytotoxic activity and relatively low toxicity toward normal cells suggests that gallium II phthalocyanine-based formulations may possess a therapeutically advantageous safety profile.

The antibacterial findings provide further evidence of the multifunctional nature of these formulations. At low concentrations, antibacterial activity against *Escherichia coli* was limited, with measurable inhibition observed primarily for chitosan-loaded formulations. The enhanced performance of chitosan-containing systems is consistent with the established antimicrobial properties of chitosan, which can interact with negatively charged bacterial membranes, increase membrane permeability, and facilitate delivery of encapsulated agents [25,86,87,88,89,90,91]. At higher concentrations, all formulations exhibited concentration-dependent antibacterial activity, with gallium II phthalocyanine again demonstrating the strongest response. The superior antibacterial performance of gallium-containing systems may be explained by disruption of bacterial iron acquisition pathways, a mechanism analogous to its effects in cancer cells. As iron is essential for bacterial metabolism and replication, gallium can function as a non-functional iron mimic, interfering with iron-dependent enzymatic processes and impairing bacterial survival [89]. Phthalocyanine-encapsulated nanoparticles exhibited pronounced light-independent anticancer and antibacterial activity [25], extending their utility beyond photodynamic therapy. Complex formation strategy enhanced stability, cellular uptake, and passive targeting, while biological effects were driven by redox disruption, mitochondrial dysfunction, and membrane destabilization [90]. These results establish phthalocyanine-based nanoplatforms as promising dual-function agents for oncology and antimicrobial applications [91].

The observation of biological activity in the absence of light activation represents one of the most significant aspects of this work. Most phthalocyanine-based studies focus primarily on photodynamic mechanisms, whereas the current findings suggest that appropriately engineered metallo phthalocyanine formulations can retain substantial biological activity under dark conditions [20,21,22]. This characteristic could potentially overcome some of the major limitations of photodynamic therapy, including restricted light penetration, dependence on external irradiation, and reduced effectiveness against deep-seated tumors. Consequently, these formulations may represent a new generation of phthalocyanine-based therapeutics capable of functioning through both photoactivated and non-photoactivated pathways.

For better drug delivery, the metal phthalocyanines were loaded into different carriers. LDL particles selectively target cancer cells due to the overexpression of LDL receptors on tumor cells, facilitating receptor-mediated uptake and enhanced intracellular drug delivery [3,34,92]. Also, PEGylation improves nanoparticle stability and prolongs systemic circulation, enabling passive accumulation within tumor tissues through the enhanced permeability and retention effect [93]. In addition, chitosan nanoparticles exhibit enhanced affinity toward cancer cells due to electrostatic interactions with negatively charged cell membranes and increased uptake under acidic tumor microenvironment conditions, and this was proved by the MTT assay [3,34,94].

In sum, Ga phthalocyanine showed potent anticancer and antibacterial activities because of its porosity surface, nanosized particles, and metal effect, which cause cell disruption without light activation [9,24,88,95]. In short, this study demonstrates that gallium (Ga II) phthalocyanine can efficiently enter cells when encapsulated within carriers, highlighting the critical role of complex formation in enhancing cellular uptake.

Despite these promising findings, several limitations should be acknowledged. First, the study was restricted to in vitro cancer and bacterial models, and therefore the in vivo pharmacokinetics, biodistribution, toxicity, and therapeutic efficacy remain to be established. Second, direct mechanistic investigations of ROS generation, apoptosis induction, mitochondrial dysfunction, and iron-metabolism disruption were not performed and should be included in future studies to confirm the proposed mechanisms. Third, only a single Gram-negative bacterial strain was evaluated, and broader antimicrobial testing against clinically relevant Gram-positive and multidrug-resistant pathogens would provide a more comprehensive assessment of antibacterial potential. Finally, quantitative measurements of loading efficiency, drug release kinetics, and receptor-mediated uptake would further strengthen understanding of the relationship between formulation properties and biological activity.

Overall, the present study demonstrates that encapsulated metallo phthalocyanines, particularly gallium II phthalocyanine, possess significant light-independent anticancer and antibacterial activity. The combination of favorable physicochemical characteristics, enhanced efficacy following complex formation, strong selectivity toward cancer cells, and dual therapeutic functionality highlights gallium II phthalocyanine complex formation as promising candidates for future translational development. Further mechanistic and in vivo investigations are warranted to validate these findings and advance this platform toward clinical application.

## 4. Materials and Methods

### 4.1. Chemicals

Sigma-Aldrich (Burlington, MA, USA) provided all the chemicals utilized for synthesis, anticancer and antibacterial research, and no additional preparation was required such as 5,6-dichloro-2,3-dicyanopyrazin, 2,6-dichlorophenol, acetonitrile, anhydrous potassium carbonate, quinoline, hexane, ethyl acetate, MgCl_2_, GaCl_2_, pentanol, DBU, Copper acetate, DMSO, polyethylene glycol (PEG 202398-Sigma Aldrich), chitosan powder (low-molecular-weight 448869—Sigma Aldrich), LDL (LDL serum-S5519), DMEM media, FBS, penicillin/streptomycin, 1% L-glutathione, PBS, and the WST-1 assay kit (ab65473, Abcam—Sigma Aldrich). The tested organism *E. coli* (strain SL06/16-1 16S ribosomal RNA gene with accession number KX279366) was obtained from the Department of Biology, College of Science, Kuwait University.

### 4.2. Instruments

All spectral analysis was carried out at Kuwait University’s Research Sector Project Unit (RSPU). FAB-MS (Thermos-6000 by Thermo Fisher Scientific, (Thermos LLC, Wiesbaden, Germany), a high-resolution gas chromatograph mass spectrometer–double focusing (GC-MS) DFS model, was used to quantify the samples’ molecular weight. A Bruker DPX 600 NMR spectrophotometer (Bruker, Billerica, MA, USA) was used to collect ^1^H nuclear magnetic resonance (NMR) spectra at 600 MHz in DMSO and CDCl_3_. A UV–Vis spectrophotometer performed optical spectroscopy, a technique used to study how light interacts with matter in the ultraviolet (UV) and visible (Vis) regions of the electromagnetic spectrum (UVVIS NIR-AGILENT CARY 5000). Elemental analysis (CHNS) was carried out using an elemental uni-cube superuser.

### 4.3. Synthesis of the Metal Phthalocyanine Agent and Characterization

Previously, we synthesized and fully characterized Mg and Cu phthalocyanine agents in our laboratories at Kuwait University. The process of creating 5,6-dichloro pyrazine-2,3-dicarbonitrile (**3**) is described in [96].

**Scheme 1:**
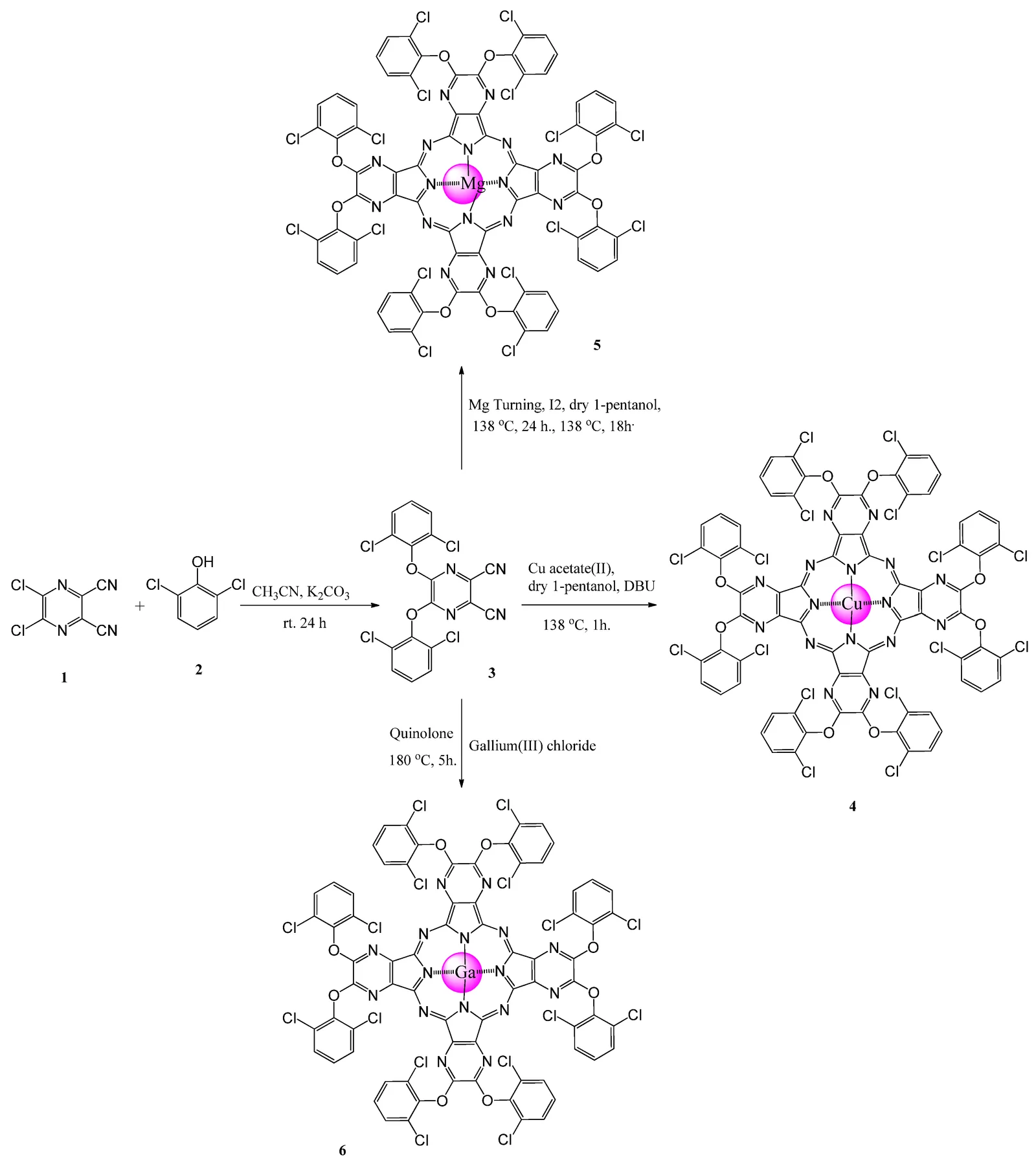
Schematic presentation of the synthesis procedures to obtain the metal phthalocyanine agents (Ga II, Cu II, Mg II).

Ga phthalocyanine production started as shown in Scheme 1, by stirring a mixture of 5,6-dichloropyrazine-2,3-dicarbonitrile (1) (1 equiv.) with 2,6-dichlorophenol (2) (2.1 equiv.) in 10 mL of dry acetonitrile solvent and anhydrous potassium carbonate (5 equiv.) as catalyst. The reaction mixture was stirred at room temperature in a nitrogen environment for 24 h. Then, 5,6-bis(2,6-dichlorophenoxy)pyrazine-2,3-dicarbonitrile (**3**) was obtained by reducing the reaction solvent by evaporation, washing the crude product many times with distilled water, and then air drying it. After that, we heated a mixture of compound (**3**) (500 mg, 1.10 mmol) with gallium chloride (44 mg, 0.276 mmol), and quinoline (3 mL) to 180 °C in a nitrogen environment for five hours. Then, nitrobenzene (2.00 mL) was added. After two hours of continuous heating, the temperature was lowered to 120 °C. Following cooling, an n-hexane: ethyl acetate (1:1, *v*/*v*) solvent mixture was added as an eluent in column chromatography on silica gel to purify the crude product known as (2,3,9,10,16,17,23,24-Octakis(2,6-dichlorophenoxy)azaphthalocyaninato Gallium).

Melting point: N300 °C; yield: 100 mg (19.30%); Ms.: 1864.844 (M+), 1519.705, 1034.498. Elemental analysis Calc. (%) for C_72_H_24_Cl_16_N_16_O_8_Ga: C, 46.05; H, 1.29; N, 11.93; Found: C, 46.11, H, 1.30; N, 11.94; ^1^H NMR (DMSO d6, 600 MHz) δ/ppm = 7.39–7.95 (m, 24 H) as shown in the Supplementary Materials.

It is important to note that ^13^C NMR spectra could not be obtained for gallium phthalocyanine due to severe line broadening induced by the quadrupolar nature of the gallium nucleus and the strong Ga–N coordination within the macrocyclic framework, which leads to rapid relaxation of nearby carbon nuclei and loss of spectral resolution. In addition, although gallium phthalocyanine is diamagnetic, the quadrupolar nature of the gallium nucleus (^69^Ga and ^71^Ga, I = 3/2) induces rapid relaxation and severe line broadening of nearby ^13^C nuclei, rendering the ^13^C NMR signals undetectable [97].

### 4.4. UV–Visible Spectroscopic Analysis

A UV–Vis spectrophotometer provides information about the electronic transitions in molecules, especially conjugated systems like interactions of phthalocyanines with light in UV–Vis regions [55,98]. It helps confirm the formation of the compound and its structural features. In a UV–Vis spectrophotometer, light from a deuterium (UV) or halogen (Vis–NIR) source passes through sample and reference cuvettes. The sample absorbs specific wavelengths, and the transmitted light is measured by a photomultiplier tube (PMT). Absorbance is proportional to concentration according to the Beer–Lambert law, producing a spectrum across the UV and visible regions [9,99].

### 4.5. TGA-Based Thermal Stability Assessment

Thermogravimetric analysis (TGA) offered insights into the thermal stability and water content of the metal phthalocyanine agents. Also, TGA allows comparison of thermal stability between materials, evaluation of effect of additives, coatings, or encapsulation, and prediction of material performance under heat. Approximately 5 mg of each agent was placed in a platinum pan and heated under a nitrogen atmosphere from 25 to 800 °C for 10 min. The measured weight reduction and the computed complex composition were clearly correlated, according to the results [3,60].

### 4.6. EDX Characterization and SEM Imaging of the Metal Phthalocyanine

The surface morphology and elemental composition of the synthesized materials were characterized using scanning electron microscopy (SEM) coupled with energy-dispersive X-ray spectroscopy (EDX, JEOL, Tokyo, Japan). EDX is a powerful analytical technique that provides both qualitative and quantitative information regarding the elemental composition and distribution of materials. When integrated with SEM, it enables localized elemental analysis of specific regions and surface features, allowing comprehensive physicochemical characterization of the samples [60]. SEM imaging was performed using a JEOL CarryScope JSM-5700 scanning electron microscope, while elemental analysis was conducted using a JEOL Flash 1400 EDX detector [9,24].

SEM analyses were carried out at an accelerating voltage of 20.0 kV, and micrographs were obtained at various magnifications (×50, ×100, ×130, ×200, ×250, ×300, ×350, ×450, ×500, ×600, ×650, and ×750). Samples prepared at concentrations of 20, 50, 100, and 500 µM were examined to evaluate their morphological characteristics. The acquired micrographs were used to assess particle size, shape, surface texture, roughness, porosity, aggregation behavior, and the presence of structural features such as cracks or fissures. The combined SEM–EDX approach enabled correlation between surface morphology and elemental composition, providing valuable insight into the structural properties of the investigated materials [60].

Prior to analysis, powder samples were mounted onto aluminum stubs using double-sided conductive adhesive tape to ensure adequate sample fixation. The mounted specimens were subsequently coated with a thin layer of gold using a Leica EM ACE200 (Germany by Leica Microsystems, Wetzlar, Germany) sputter coater to improve electrical conductivity and minimize charging effects during electron beam exposure. All SEM and EDX analyses were conducted at the Research and Scientific Projects Unit (RSPU), and the resulting images and spectra were used to evaluate the morphological and elemental characteristics of the synthesized materials [68].

### 4.7. Complex Formation of Metal Phthalocyanines

Low-density lipoprotein (LDL; Sigma-Aldrich, S5519), polyethylene glycol (PEG; Sigma-Aldrich, CAS No. 25322-68-3, average molecular weight Mn 400, hydroxyl-terminated), and chitosan (Chi; Sigma-Aldrich, CAS No. 9012-76-4, low molecular weight) were used as received without further preparation. To prepare the chitosan solution, acetic acid was diluted in distilled water to obtain 1% *v*/*v*. Subsequently, one gram of chitosan powder was gradually added to 100 mL of the solution under continuous stirring at 50–60 °C until a homogeneous and transparent solution was obtained. The pH of the chitosan solution was adjusted to approximately 5.5–6.0 using 0.1 M NaOH to improve polymer stability and facilitate particle aggregates formation. The resulting LDL, PEG, and chitosan preparations were used as particle carriers in the liquid phase.

**Figure 3:**
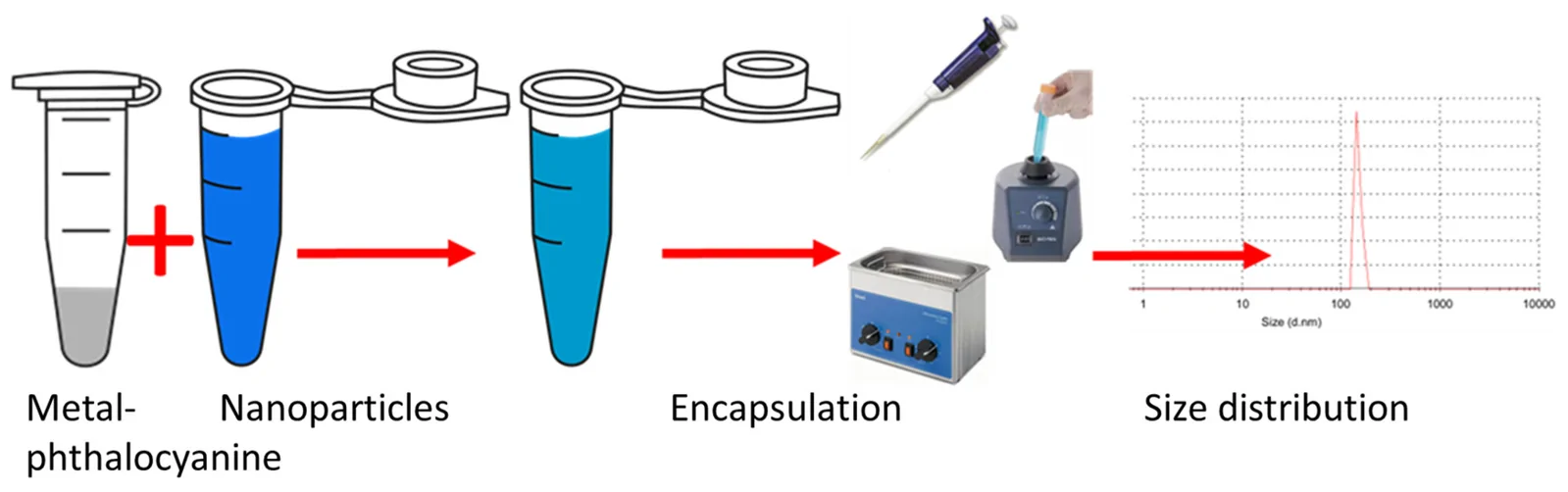
Schematic representation of nanoparticle preparation and complex formation: the metal phthalocyanine solution is mixed and pipetted into the particle matrix, followed by sonication for one hour. The resulting particles are analyzed using DLS to determine their size distribution.

Next, for complex formation, 50 µL of each metal phthalocyanine was incorporated and mixed with 250 µL particles using pipetting, vortex mixing, and sonication at a 1:5 agent-to-particle ratio (1:5 *v*/*v*), as shown in Figure 3. The mixtures were initially homogenized by pipetting and vortex mixing for 5 min, followed by sonication in an ultrasonic bath for 45–60 min at room temperature (28 ± 2 °C) to promote uniform dispersion and enhance interactions between the phthalocyanine molecules and the carrier systems. The resulting suspensions were further homogenized using a Polytron dispersing and mixing device (Kinematica AG, Lucerne, Switzerland) and then incubated overnight (24 h) at 4 °C to facilitate complex formation reorganization and efficient loading of the metal phthalocyanines. The same procedure was applied to all investigated agents, while unloaded LDL, PEG, and chitosan particles were prepared under identical conditions and used as controls [3,9,28,29,100]. Following preparation, all formulations were stored in sterile, amber-colored containers at 4 °C to protect the samples from light-induced degradation and maintain physicochemical stability. Samples were kept under refrigerated conditions and used within one week of preparation to minimize potential changes in particle size, loading efficiency, and biological activity. Prior to characterization or biological assays, the formulations were gently vortexed to ensure uniform dispersion.

### 4.8. Dynamic Light Scattering Measuring the Particles’ Size

Particle size distribution and zeta potential measurements were determined using a Malvern Zetasizer Nano ZS (Malvern Panalytical Ltd., Great Malvern, Worcestershire, UK), which operates based on dynamic light scattering and electrophoretic light scattering (ELS) principles. DLS was employed to determine the hydrodynamic diameter and size distribution of the nanoparticles, while ELS was used to evaluate surface charge (zeta potential), an important indicator of colloidal stability. The instrument is capable of measuring particle sizes ranging from 0.3 nm to 10 µm and incorporates automatic temperature control to ensure measurement accuracy and reproducibility. All analyses were performed at the Research Sector Projects Unit (RSPU), Kuwait University. Prior to measurement, 100 µL of free nanoparticles (LDL, PEG, and chitosan) or metal-phthalocyanine-loaded particle formulations were diluted with deionized water to obtain an appropriate scattering intensity and prevent multiple scattering effects. The samples were gently vortexed and subsequently sonicated in an ultrasonic bath for 5 min at room temperature (25 ± 2 °C) to ensure complete dispersion and minimize particle aggregation. Following sonication, the suspensions were transferred into disposable polystyrene cuvettes for particle size measurements. All measurements were conducted at pH 7.0, and samples were allowed to equilibrate within the instrument for 2 min before data acquisition.

Particle size measurements were performed at a scattering angle of 173° (backscatter detection mode) and a controlled temperature of 25 °C. For each sample, three independent measurements were obtained, with each measurement consisting of multiple runs to ensure reproducibility. The average hydrodynamic diameter and polydispersity index (PDI) values were recorded and expressed as mean ± standard deviation (SD). These parameters were used to evaluate particle size distribution, dispersion homogeneity, and colloidal stability before subsequent physicochemical and biological analyses [3,60,101].

### 4.9. Cell Culture

Dulbecco’s Modified Eagle Media (DMEM-D0822, Sigma Aldrich and Merck, Darmstadt, Germany) was supplemented with 10% FBS, 1% penicillin/streptomycin, and 1% L-glutathione. FBS was inactivated in a water bath at 37 °C for 30 min before being used. All reagents were purchased from Sigma-Aldrich and ready for direct use without further preparation [9,24].

### 4.10. Assessment of Cell Proliferation Induced by Metal Phthalocyanine Agents

The cytotoxic effects of the synthesized compounds and their nanoparticle formulations were evaluated using the MTT assay, a widely employed colorimetric method for assessing cell viability and proliferation based on mitochondrial metabolic activity in viable cells [68,102]. Human liver carcinoma (HepG2) and human breast carcinoma (MDA-MB-453) cell lines were cultured under standard conditions and maintained in complete growth medium supplemented with fetal bovine serum and antibiotics. Cells were seeded into 96-well microplates at a density of 1 × 10^5^ cells/well and incubated overnight at 37 °C in a humidified atmosphere containing 5% CO_2_ to allow cell attachment and stabilization. The plates were then incubated for 2 h at 37 °C to allow viable cells to reduce the yellow tetrazolium salt into insoluble purple formazan crystals, followed by the addition of 100 µL of MTT solution after the removal of the old media and incubated for two hours. Next, the addition of 100 µL dimethyl sulfoxide (DMSO) was accompanied by ten minutes of shaking to ensure complete solubilization. Absorbance was subsequently measured at 450 nm using a microplate reader (Gen5, Molecular Devices, Dasman Diabetes Institute, Kuwait city, Kuwait). Cell viability was calculated relative to untreated control cells, and all treatments were performed in quadruplicate. Results are expressed as mean ± standard deviation (SD) from independent experiments [103].

### 4.11. Statistical Analysis

Statistical analyses were performed using GraphPad Prism version 9.0 (GraphPad Software, San Diego, CA, USA). All experiments were conducted in quadruplicate, and the results are presented as mean ± standard error (SE). Comparisons between experimental groups were performed using the appropriate statistical tests available within GraphPad Prism (nonlinear regression analysis). Differences were considered statistically significant when the *p*-value was less than 0.005. Statistical analysis was used to evaluate the reproducibility, reliability, and significance of the observed differences in physicochemical properties and biological activities among the tested formulations [68,104].

### 4.12. Assessment of Antibacterial Activity Targeting Gram-Negative Bacteria

Gram-negative bacteria *E. coli* (strain SL06/16-1 16S ribosomal RNA gene with accession number KX279366) was a gift obtained from the Department of Biology, College of Science, Kuwait University. Antibacterial activity of the synthesized metal phthalocyanines and their particle formulations was evaluated against *Escherichia coli* using agar well diffusion assays in accordance with Clinical and Laboratory Standards Institute (CLSI) guidelines adopted by Kuwait University [60,105,106,107]. All microbiological procedures were performed under strict aseptic conditions in a laminar flow cabinet or near a Bunsen burner flame to minimize contamination. Nutrient agar (NA, Sigma Aldrich) plates were prepared according to Difco manufacturer instructions and allowed to stabilize for 48 h before use. Bacterial inocula were prepared by transferring freshly cultured colonies into nutrient broth and adjusting the suspension to approximately 5 × 10^5^ CFU mL^−1^.

For antibacterial testing, bacterial suspension was uniformly spread onto the surface of sterile nutrient agar plates using a sterile cotton swab to obtain a confluent bacterial lawn. In parallel, agar well diffusion assays were performed by creating uniform wells in the agar using a sterile cork borer, followed by the addition of 100 µL of each metal phthalocyanine formulation into the respective wells. The plates were incubated at 37 °C for 24 h, after which antibacterial activity was assessed by measuring the diameter of the inhibition zones surrounding the disks or wells. The minimum inhibitory zones were determined by measuring the visible growth inhibition using the ruler.

To investigate concentration-dependent antibacterial effects, the metal phthalocyanines and their encapsulated formulations were tested at different concentrations. The diameters of both clear and total inhibition zones were measured using a calibrated ruler and recorded in millimeters (mm). All experiments were performed in triplicate, and the results were expressed as mean values. The observed inhibition zones were used as indicators of antibacterial efficacy, with larger zones corresponding to greater antimicrobial activity against the Gram-negative bacterial strain [105,106,107].

## 5. Conclusions

In conclusion, the synthesized metal phthalocyanines were successfully designed, synthesized, and thoroughly characterized. Elemental analysis by EDX confirmed the accurate composition of the synthesized agents, while SEM revealed rough and porous surface morphologies. DLS measurements verified the successful loading of the agents. Biological evaluation against HepG2 and MDA-MB-453 cancer cell lines demonstrated significant anticancer activity, particularly for gallium II phthalocyanine, which exhibited the lowest IC_50_ values and the highest overall potency. The encapsulated formulations also showed concentration-dependent antibacterial activity against *Escherichia coli*. The enhanced biological performance of gallium II phthalocyanine may be attributed to its favorable physicochemical properties and porous nanoscale structure, which can facilitate improved interactions with cellular and bacterial targets. Overall, these findings highlight gallium II phthalocyanine as a promising non-photoactivated candidate for future anticancer and antibacterial applications.

## Figures and Tables

**Table 1:** The UV–Vis absorption spectra of Cu, Mg, and Ga phthalocyanines were recorded in DMSO solvent over the wavelength range of 200–800 nm.

Agent	Electronic Absorbance Spectra	Wavelength Range (nm)
Cu Phthalocyanine	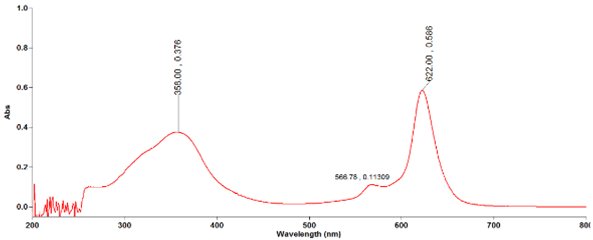	Phthalocyanine 358–622
Mg Phthalocyanine	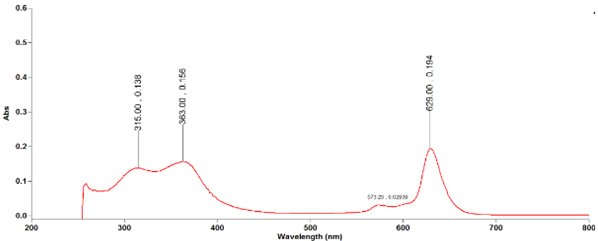	Phthalocyanine 315, 366–596, 627
Ga Phthalocyanine	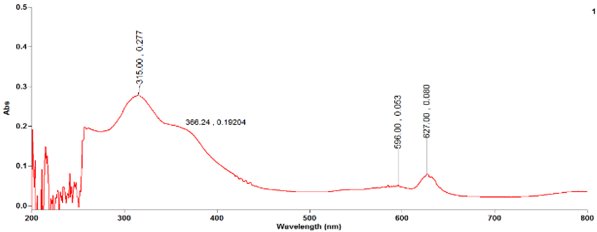	Phthalocyanine 315, 396–627

**Table 2:** Thermogravimetric assessment of phthalocyanine agents’ stability under physiological conditions.

Agent	TGA Thermogram (25–800 °C)
Cu Phthalocyanine	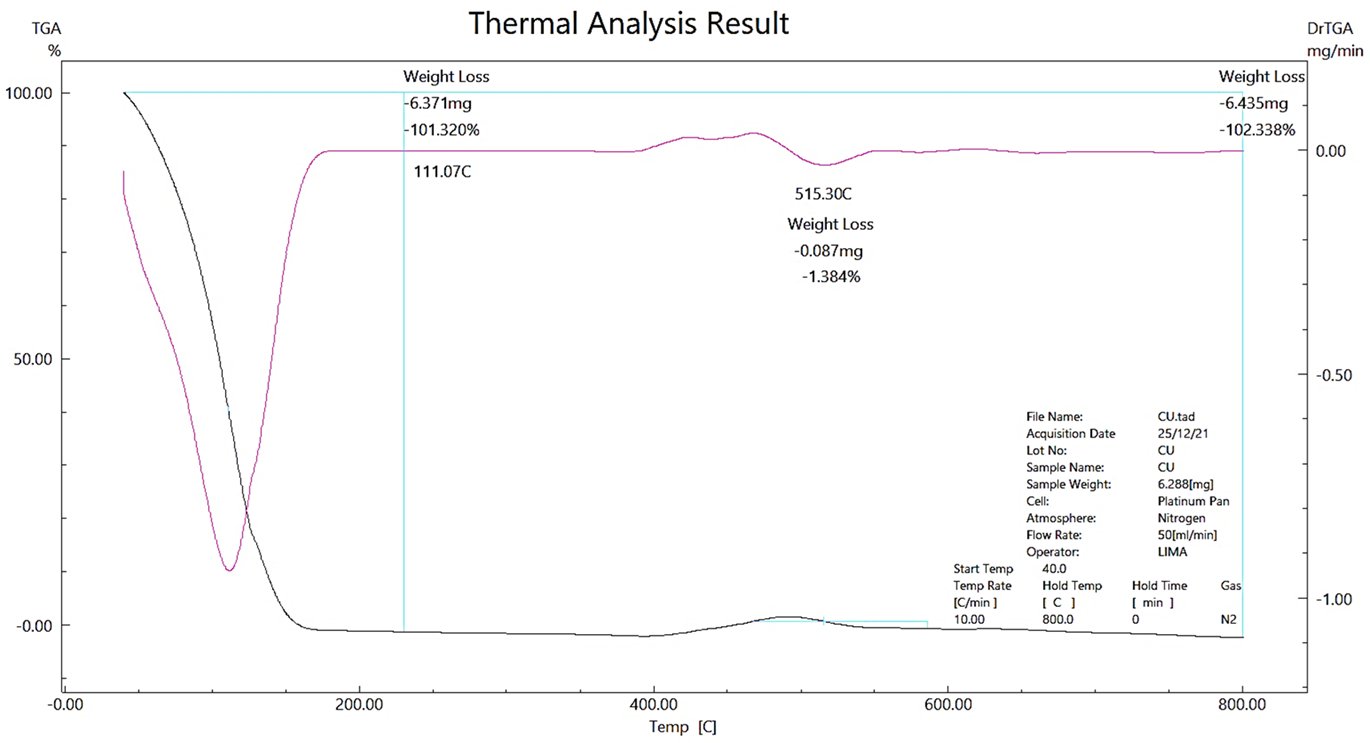
Mg Phthalocyanine	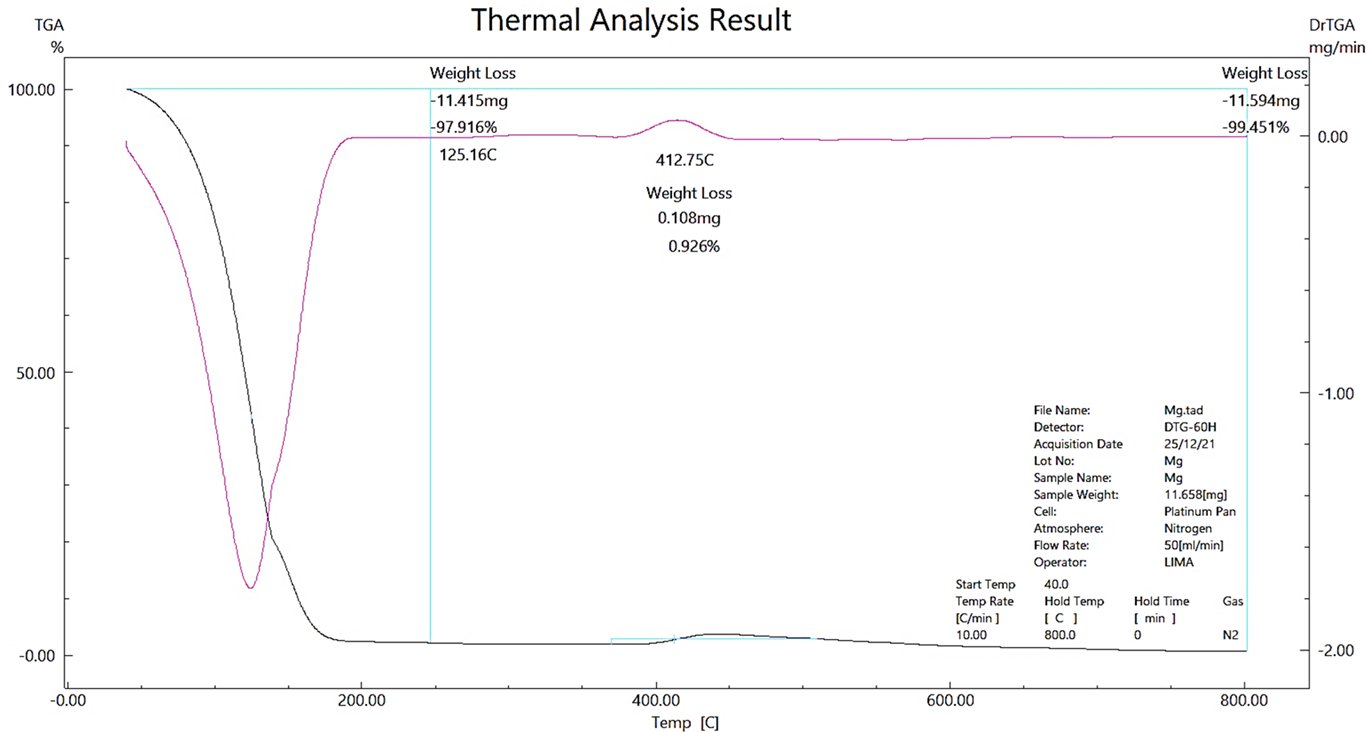
Ga Phthalocyanine	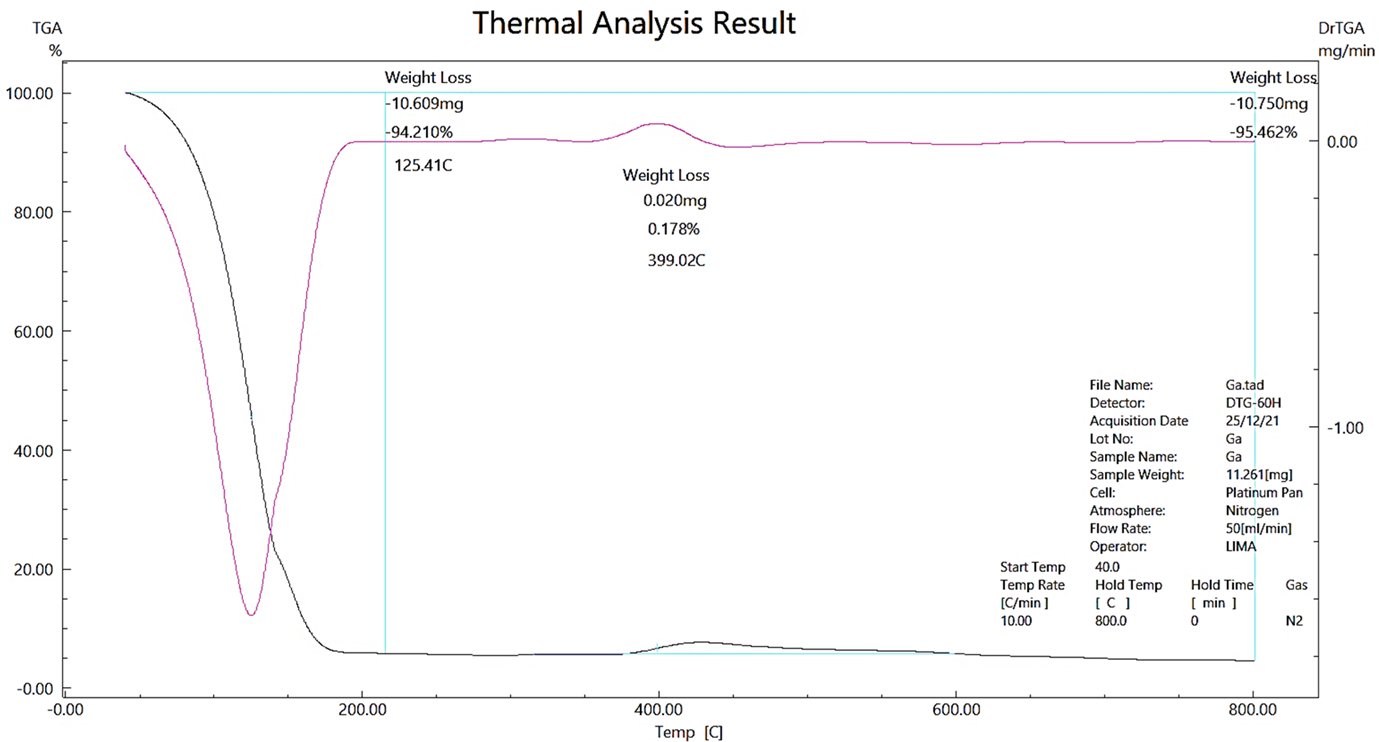

Note: 37 °C, normal body temperature; 41 °C, high body temperature = fever.

**Table 3:** EDX measurements were conducted on Cu, Mg, and Ga phthalocyanine agents to determine the elemental percentages and overall weight contribution in the selected positions of the samples.

**Cu Phthalocyanine**	**Weight %**	**Atomic %**	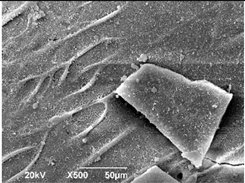
C	65.29	82.80	
Cl	23.48	10.09	
Cu	5.03	1.21	
O	6.20	5.90	
Total	100	100	
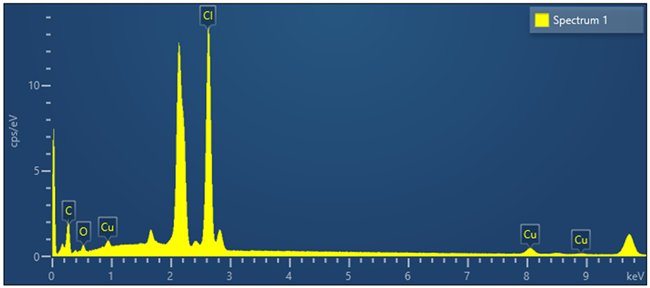			
**Mg Phthalocyanine**	**Weight %**	**Atomic %**	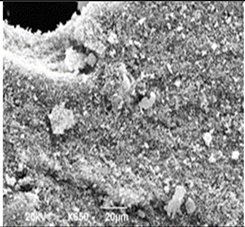
**C**	57.61	69.44	
**Cl**	16.61	6.78	
**O**	9.90	8.96	
**N**	14.01	14.48	
**Mg**	0.25	0.15	
**I**	1.61	0.18	
**Total**	100	100	
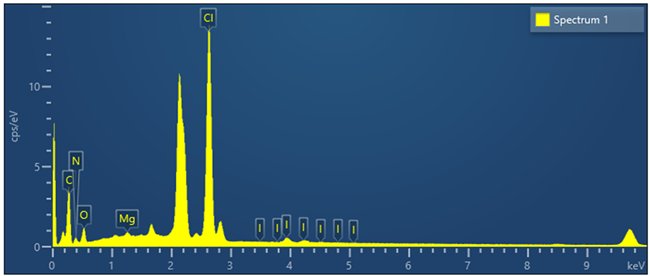			
**Ga Phthalocyanine**	**Weight %**	**Atomic %**	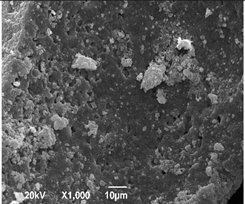
C	79.54	88.13	
O	9.83	8.18	
Cl	9.12	3.42	
Ga	1.07	0.20	
Rb	0.43	0.07	
Total	100	100	
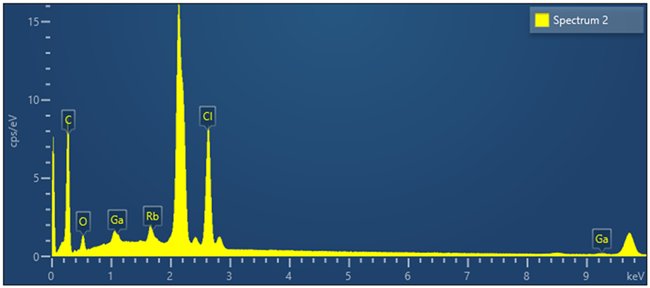			

**Table 4:** SEM micrographs for the Cu, Mg, Ga phthalocyanine at different magnifications X50–X750 at 20, 50, 100, and 500 µM (at 20 kV).

Agent/Structure	SEM Images	
Cu Phthalocyanine Nanoparticles, sharp, rough surfaces.	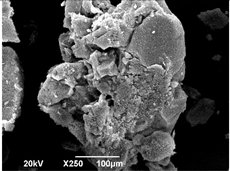	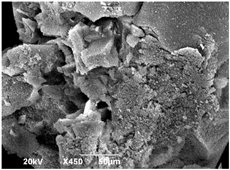
	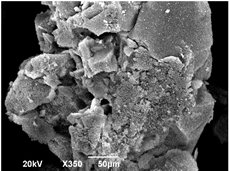	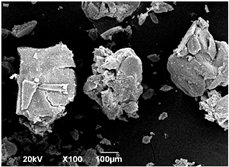
Mg Phthalocyanine Nanoparticles, sharp, rough surfaces.	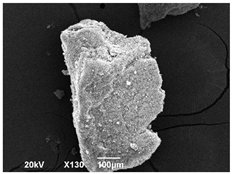	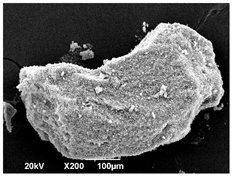
	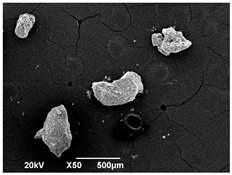	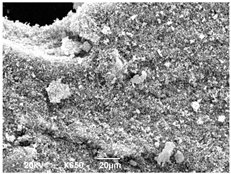
Ga Phthalocyanine Nanoparticles, porous, sheets surfaces.	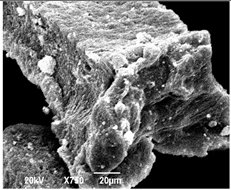	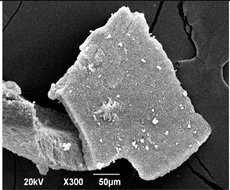
	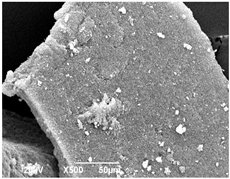	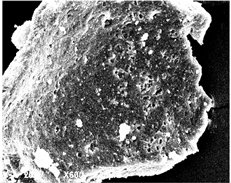

**Table 5:** DLS measurements of Cu, Mg, and Ga phthalocyanines were performed in water.

Agent	Size of Agent Before Complex Formation	Agent Size After Complex Formation with LDL Particles DPI (Intensity)	Agent Size After Complex Formation with PEG Particles DPI (Intensity)	Agent Size After Complex Formation with Chitosan Particles DPI (Intensity)
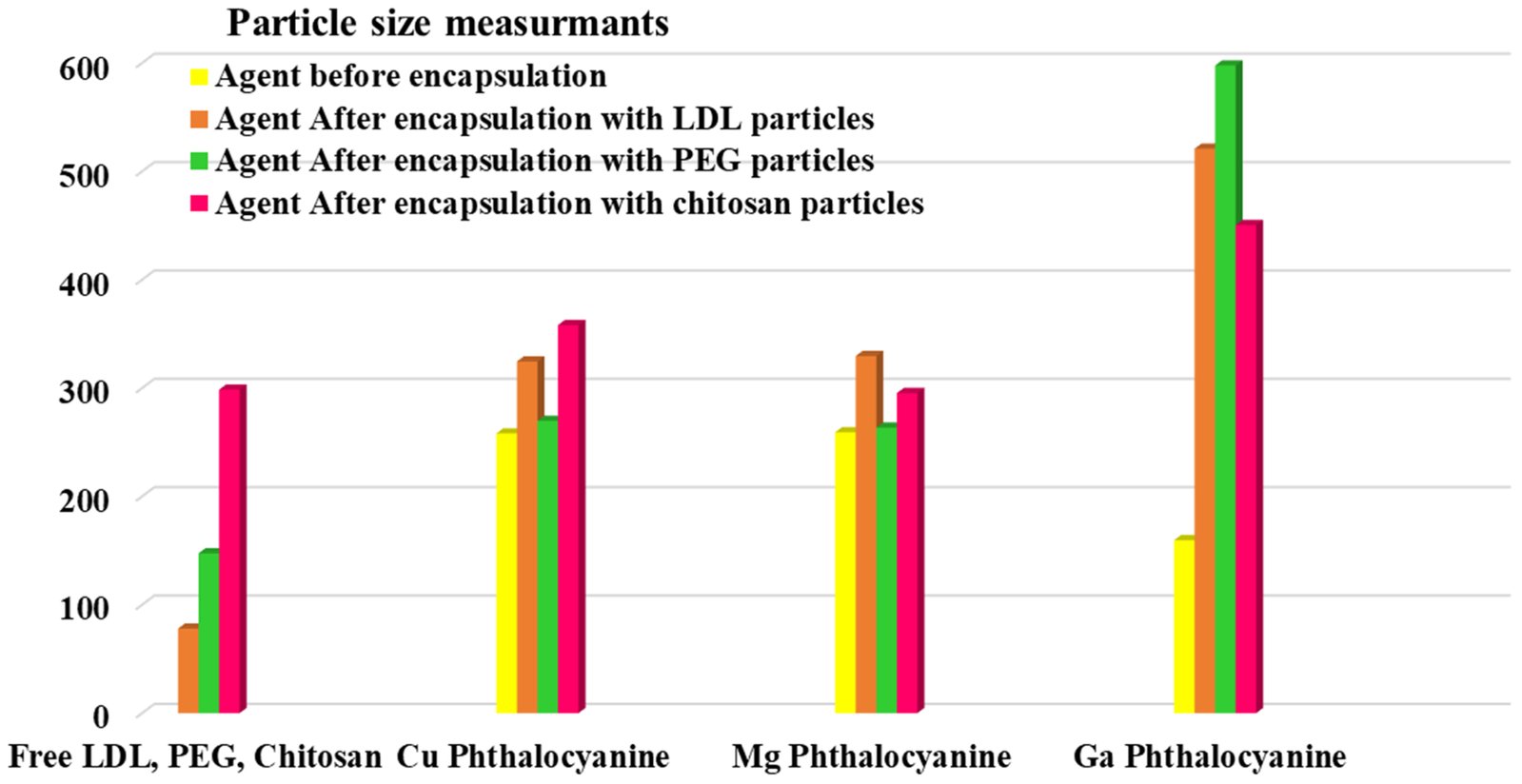				
Free LDL, PEG, Chitosan	-	78.10 ± 3.569 0.382 (49)	147.5 ± 9.748 1.000 (100)	298.5 ± 28.76 1.000 (100)
Cu Phthalocyanine	258.1 ± 64.74 0.510 (100)	324.5 ± 22.59 0.787 (100)	269.7 ± 28.42 0.397 (71)	358.1 ± 24.71 0920 (100)
Mg Phthalocyanine	259.2 ± 66.86 1.000 (82)	329.6 ± 20.60 0.932 (100)	263.4 ± 36.99 0651 (100)	295.3 ± 3.815 0.604 (100)
Ga Phthalocyanine	159.5 ± 38.11 0.482 (100)	520.9 ± 95.74 0.339 (100)	597.7 ± 94.30 0.806 (55)	450.4 ± 21.24 0.946 (100)

The particle size average diameter (nm) ± standard deviation. PDI < 0.10 (highly monodisperse). PDI 0.10–0.30 (narrow distribution). PDI 0.30–0.50 (moderate distribution). PDI > 0.50 (broad or heterogeneous distribution).

**Table 6:** The IC_50_ data of the treated breast cancer cells with Cu, Mg, Ga phthalocyanine at concentration of 25 µM.

MDA-MB-453	IC_50_ Values Agent Treatment at 25 µM	IC_50_ Values Agent Loaded LDL Treatment 25 µM	IC_50_ Values Agent Loaded PEG Treatment 25 µM	IC_50_ Values Agent Loaded Chitosan Treatment 25 µM	IC_50_ Values Agent Treatment with HEK293 at 25 µM
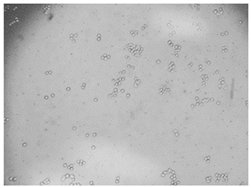 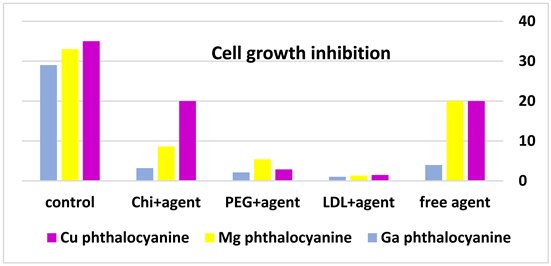					
Cu phthalocyanine	>20	1.478 ± 0.144	2.86 ± 0.113	>20	35.11 ± 0.142
Mg phthalocyanine	>20	1.319 ± 0.151	5.402 ± 0.120	8.597 ± 0.120	33.24 ± 0.514
Ga phthalocyanine *	3.965 ± 0.097	1.030 ± 0.112	2.114 ± 0.143	3.207 ± 0.104	29.36 ± 0.215

*: The most potent. LDL best drug delivery (LDL > PEG > chitosan). MDA-MB-453 = breast cancer cell line. HEK293 = healthy cells used as control. Agents loaded to carrier (1:5 ratio).

**Table 7:** The IC_50_ data of treated liver cancer cells with Cu, Mg, Ga phthalocyanine at concentration of 25 µM.

HEPG2	IC_50_ Values Agent Treatment at 25 µM	IC_50_ Values Agent Loaded LDL Treatment 25 µM	IC_50_ Values Agent Loaded PEG Treatment 25 µM	IC_50_ Values Agent Loaded Chitosan Treatment 25 µM	IC_50_ Values Agent Treatment with HEK293 at 25 µM
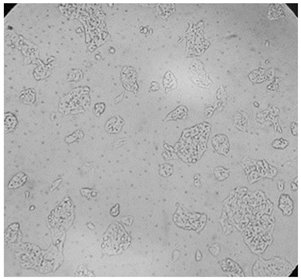 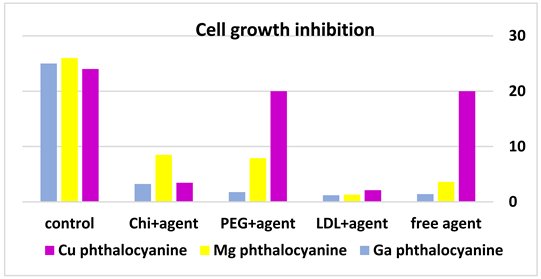					
Cu phthalocyanine	>20	2.108 ± 0.111	3.450 ± 0.109	8.610 ± 0.111	24.01 ± 0.327
Mg phthalocyanine	3.616 ± 0.110	1.296 ± 0.111	7.906 ± 0.136	8.483 ± 0.100	26.22 ± 0.429
Ga phthalocyanine *	1.401 ± 0.101	1.193 ± 0.102	1.754 ± 0.103	3.221 ± 0.130	24.32 ± 0.485

*: The most potent. LDL = best drug delivery. HEPG2 = liver cancer cell line. HEK293 = healthy cells used as control. Agents loaded to carrier (1:5 ratio).

**Table 8:** Antibacterial activity of metal phthalocyanine agents at 10 µM concentration.

Agent/*E. coli*	Free Agents (Control)	Agents Loaded with LDL 10 µM (Diameter-mm)	Agents Loaded with PEG 10 µM (Diameter-mm)	Agents Loaded with Chitosan 10 µM (Diameter-mm)
Cu phthalocyanine	Nill	Nill	Nill	1.8 *
Mg phthalocyanine	Nill	Nill	Nill	1.6 *
Ga phthalocyanine	Nill	Nill	Nill	2.2 *
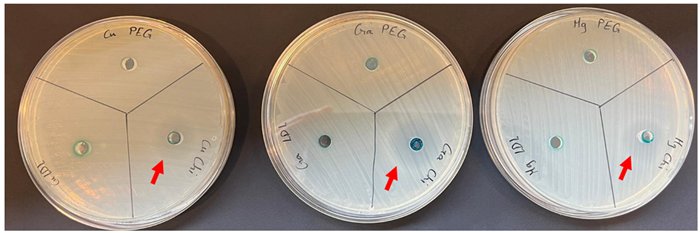				

* Weak bacterial inhibition zone, not clear zones. The results are an average of three runs. *E. coli* is prokaryotic cells (Gram-negative bacteria). The diameter was measured by ruler.

**Table 9:** The antibacterial activity of the synthesized metal phthalocyanine agents was evaluated against a representative Gram-negative bacterial strain, revealing concentration-dependent inhibitory effects even in the absence of light activation.

Encapsulated with Chitosan 50 µM Concentration		
Clear Inhibition Zone		Weak Inhibition Zone
Cu phthalocyanine	1.3 cm	3.0 cm
Mg phthalocyanine	1.2 cm	2.9 cm
Ga phthalocyanine	1.3 cm	3.0 cm
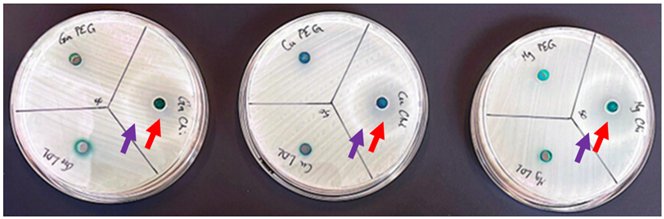		
**Encapsulated with Chitosan 70 µM**		
**Clear Inhibition Zone**		**Weak Inhibition Zone**
Cu phthalocyanine	1.7 cm	3.4 cm
Mg phthalocyanine	1.7 cm	3.2 cm
Ga phthalocyanine *	2.0 cm	3.5 cm
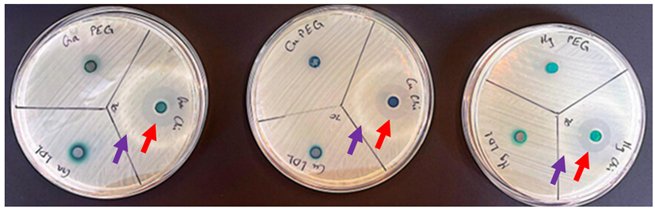		

* *E. coli*: Gram-negative bacteria, potent. Values represent inhibition zone diameters measured after treatment with chitosan-encapsulated Cu, Mg, and Ga phthalocyanines at concentrations of 50 and 70 µM using a ruler. The clear inhibition zone corresponds to the region of complete bacterial growth suppression, whereas the weak inhibition zone represents the surrounding area of partial growth inhibition. Larger inhibition zone diameters indicate greater antibacterial activity. *Escherichia coli* is a Gram-negative bacterial strain commonly used for evaluating antimicrobial efficacy. Among the tested compounds, Ga phthalocyanine exhibited the strongest antibacterial activity, particularly at 70 µM, where the largest clear and overall inhibition zones were observed.

## Data Availability

Data available within the article or its Supplementary Materials.

## Supplementary Materials

The following supporting information can be downloaded at: https://www.mdpi.com/article/10.3390/ijms27167322/s1.
