# Supplementary material for: Dual Anticancer and Antibacterial Applications of Active Particle Aggregates Containing Metal Phthalocyanines in Dark
Source: Int J Mol Sci. 2026 Aug 16;27(16):7322. doi: 10.3390/ijms27167322 (PMC13513340; doi:10.3390/ijms27167322)
Supplement: Supplementary file 1 [file ijms-27-07322-s001.zip › ijms-4487823-supplementary.pdf]

Supplementary Information

Dual Anticancer and Antibacterial Applications of Active Particle Aggregates Containing Metal Phthalocyanines in Dark

Laila Jaragh-Alhdadad [1,2,*], Moayyad Alsawah [1] and Rasheed Ahmad [2]

[1] Department of Chemistry, College of Science, Kuwait University, Sabah Al Salem University City, Shadadiya, Safat 13060, Kuwait; moayyad.alsawah@ku.edu.kw
[2] Immunology & Microbiology Department, Dasman Diabetic Institute, Dasman, Kuwait City 15462, Kuwait; rasheed.ahmad@dasmaninstitute.org
* Correspondence: laila.alhadad@ku.edu.kw

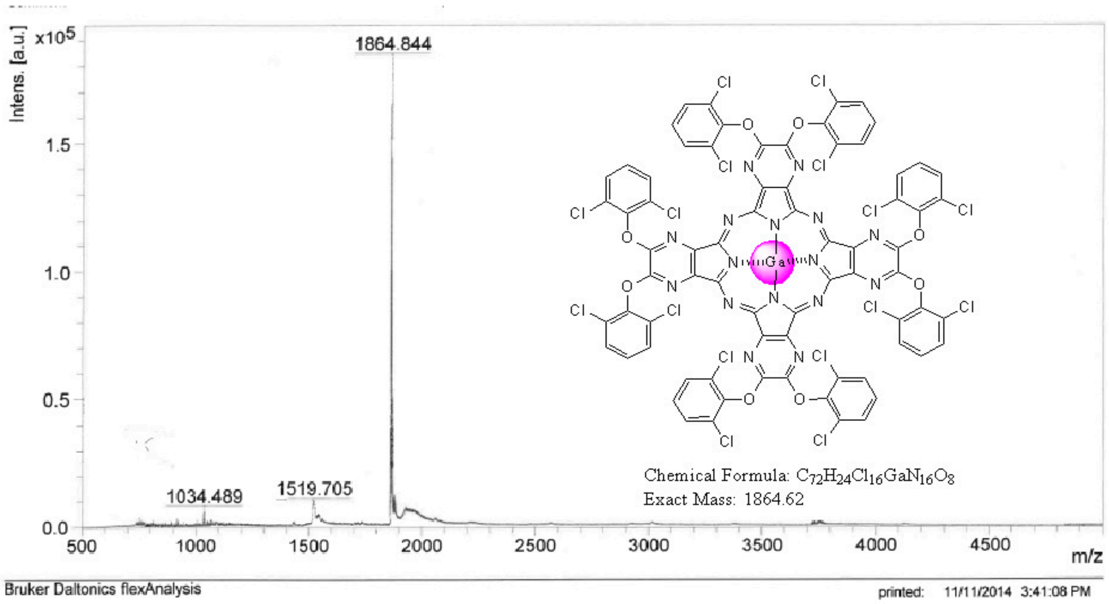

Figure S1. Mass spectrum of the Ga phthalocyanine.

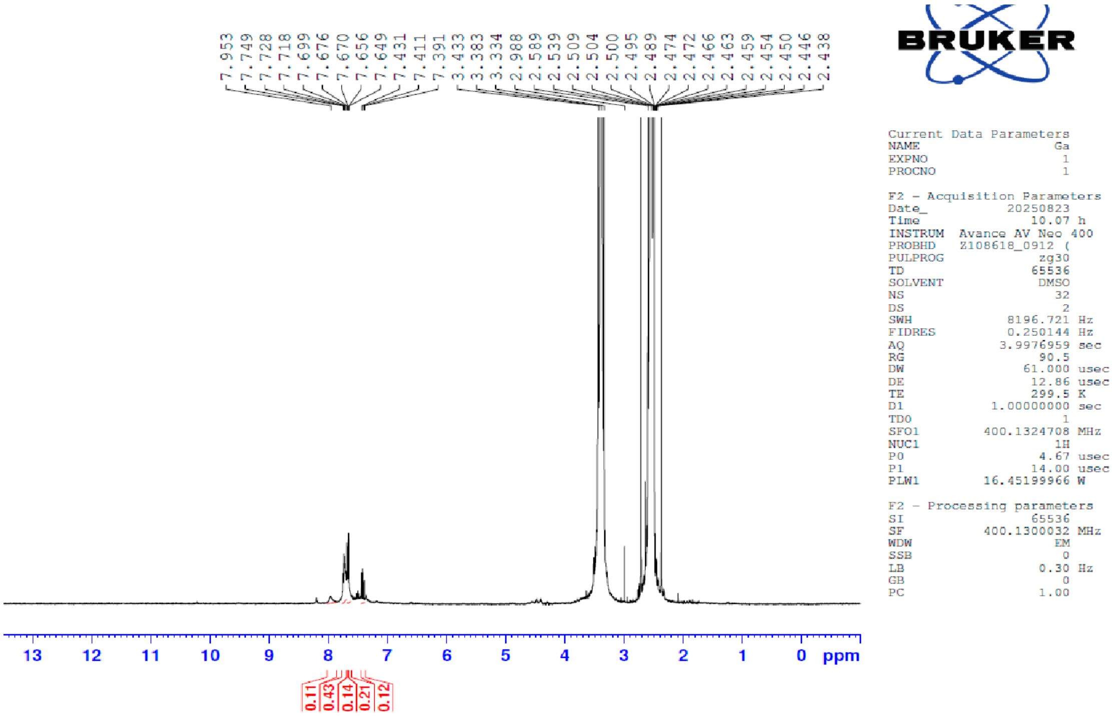

Figure S2. The ^1H NMR spectrum in DMSO for Ga phthalocyanine agent.

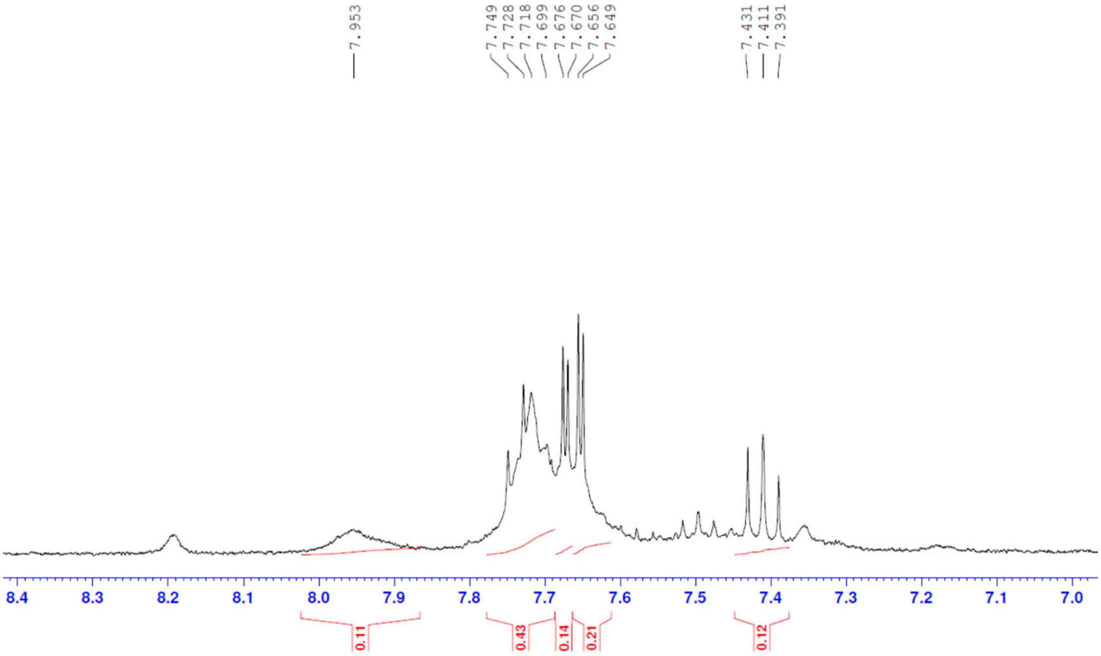

Figure S3. Detailed ^1H NMR spectra in DMSO for Ga phthalocyanine agent.

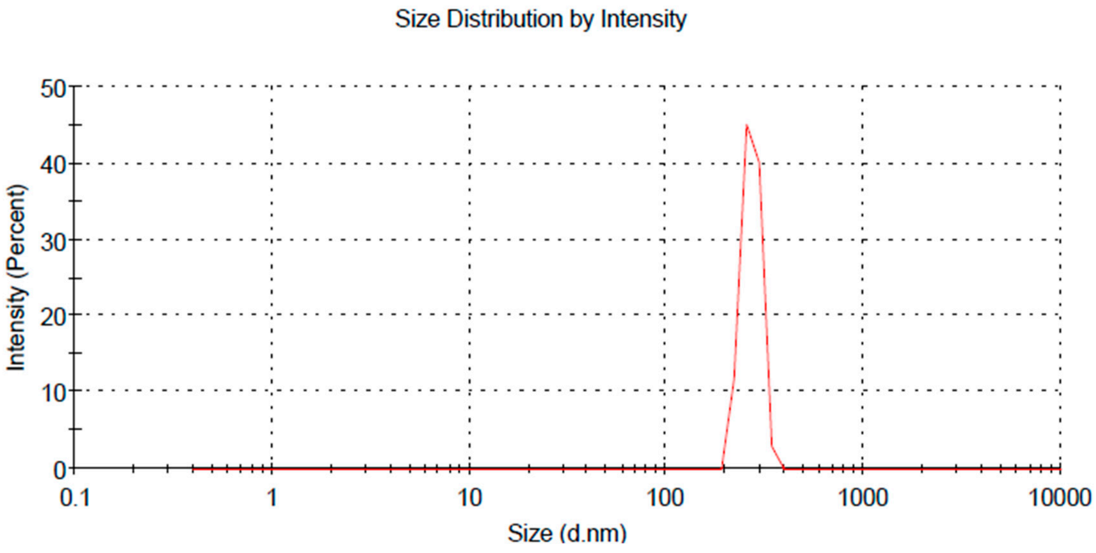

Figure S4. Cu phthalocyanine particle size.

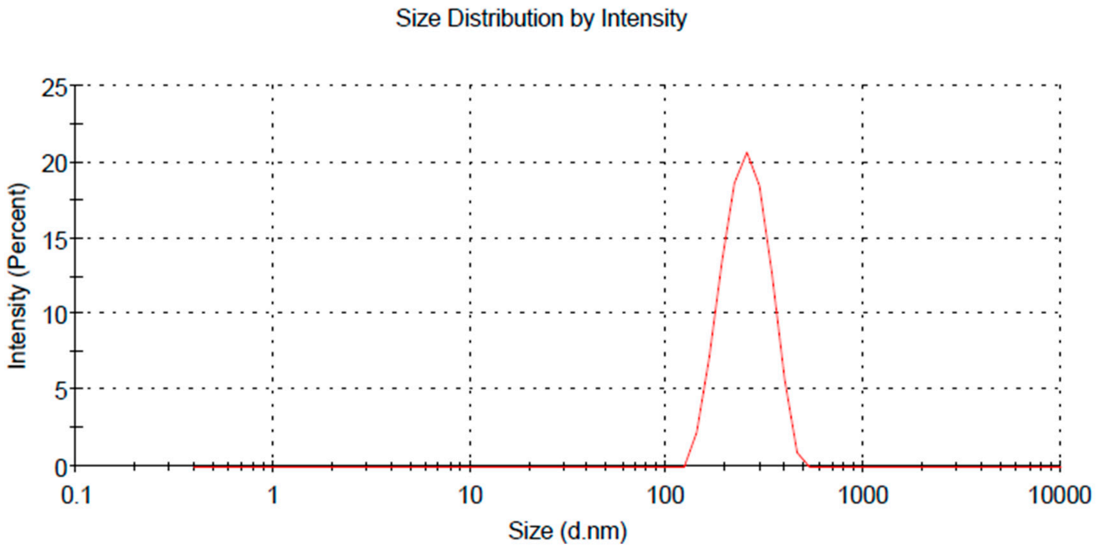

Figure S5. Mg phthalocyanine particle size.

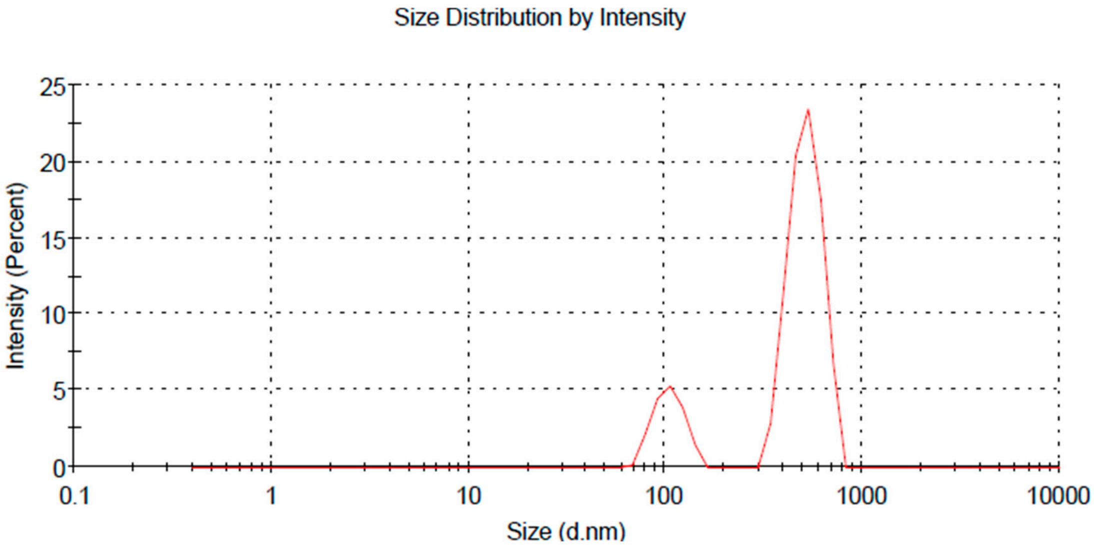

Figure S6. Ga phthalocyanine particle size.

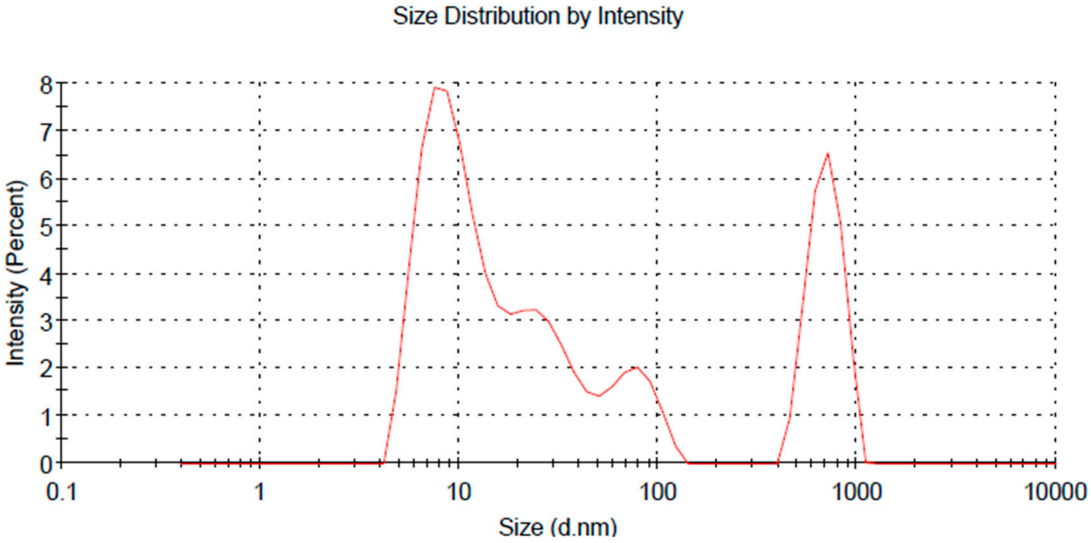

Figure S7. LDL particle size measurment.

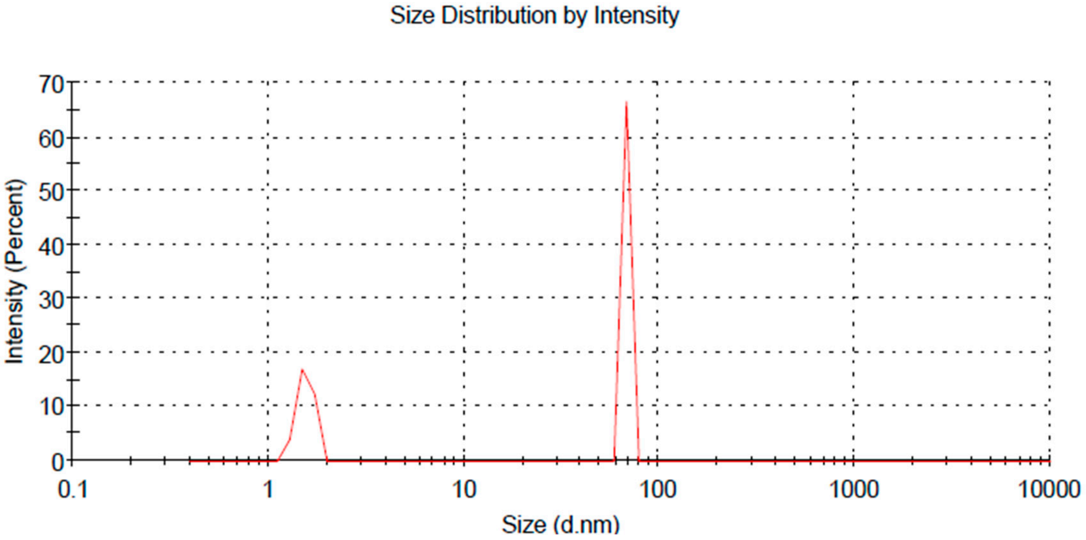

Figure S8. PEG particle size measurment.

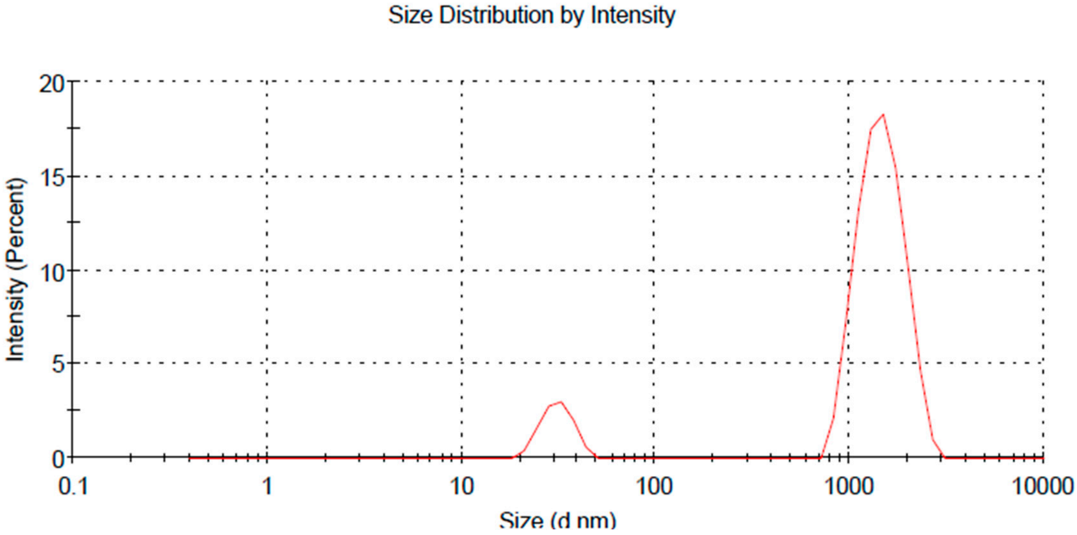

Figure S9. Chitosan particle size measurment.

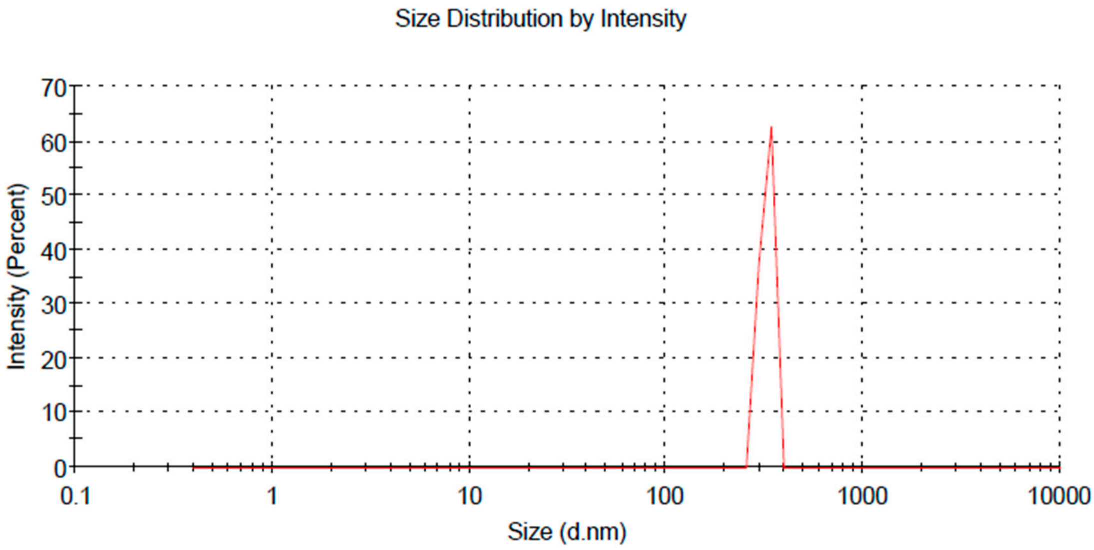

Figure S10. Cu phthalocyanine -LDL complex formation.

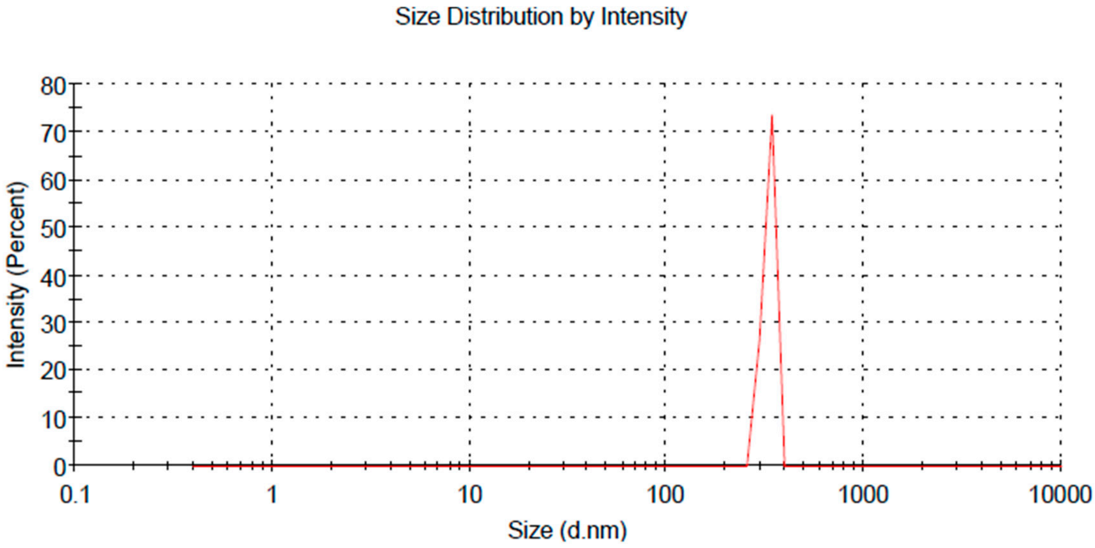

Figure S11. Mg phthalocyanine -LDL complex formation.

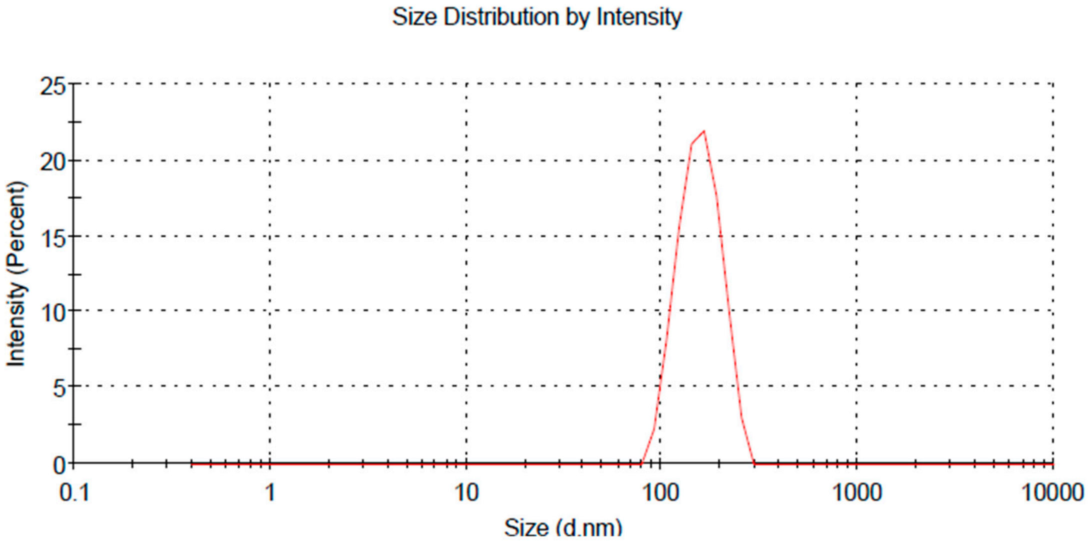

Figure S12. Ga phthalocyanine -LDL complex formation.

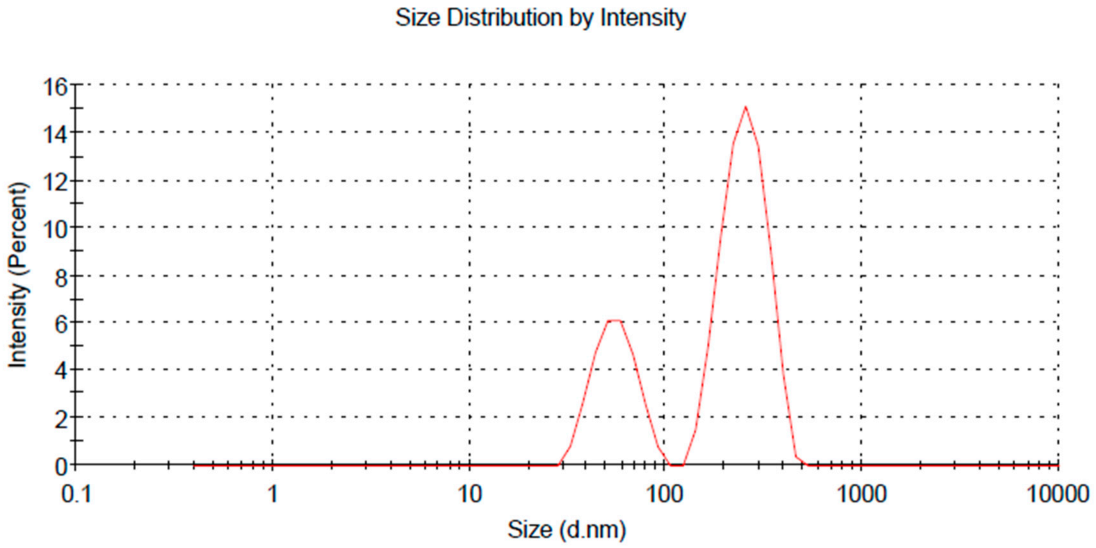

Figure S13. Cu phthalocyanine -PEG complex formation.

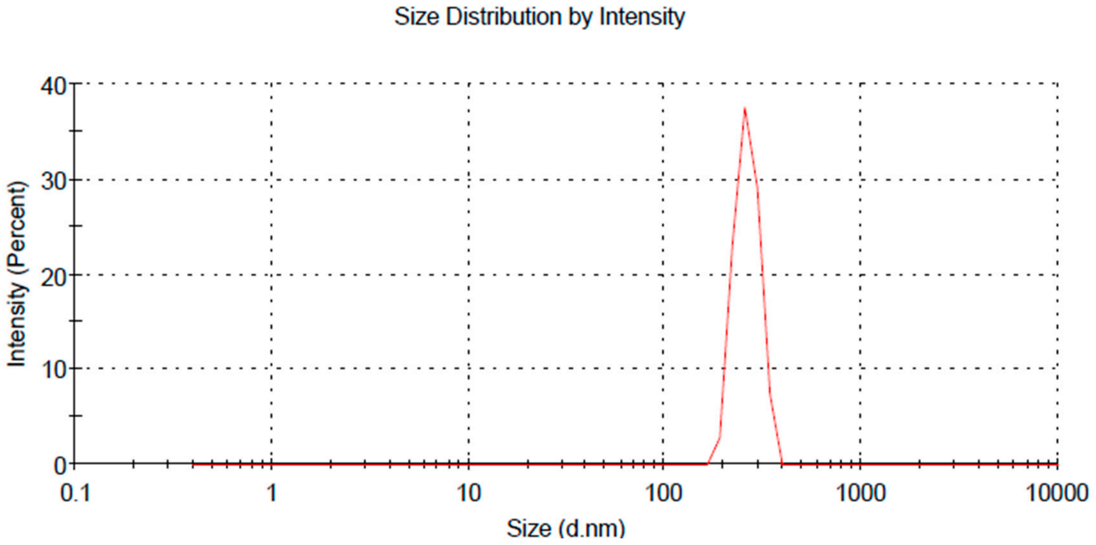

Figure S14. Mg phthalocyanine -PEG complex formation.

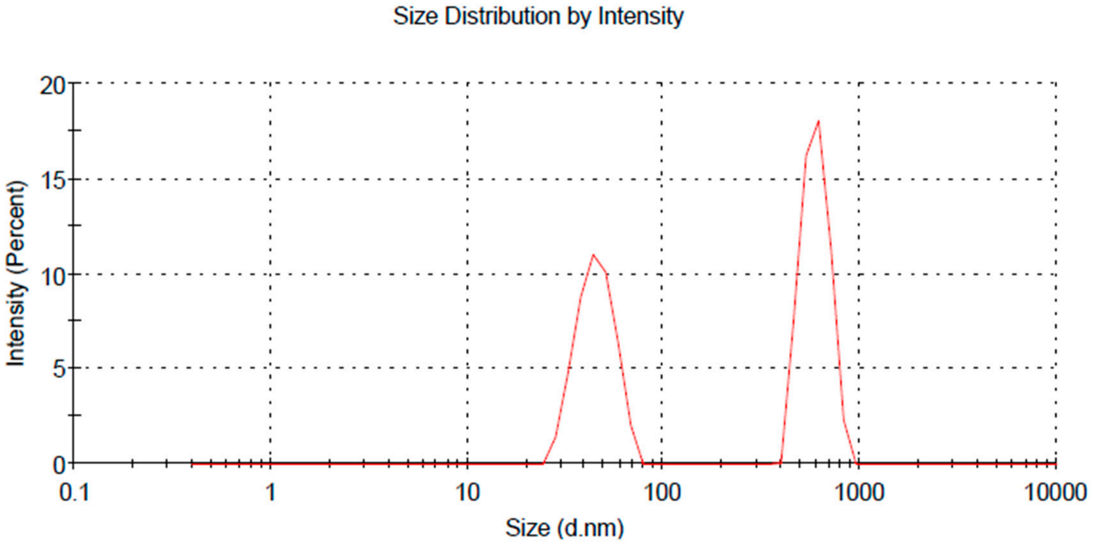

Figure S15. Ga phthalocyanine -PEG complex formation.

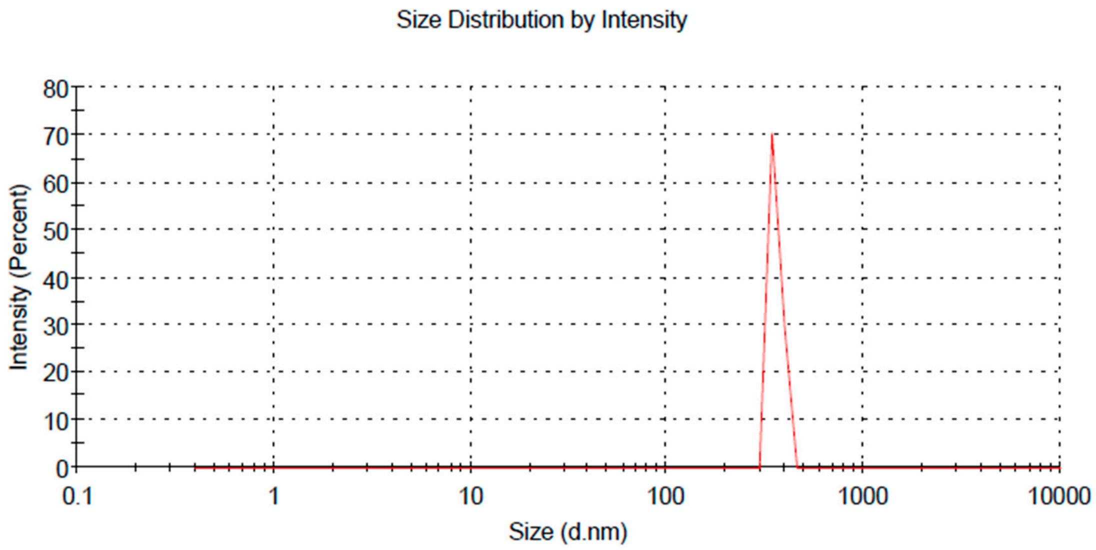

Figure S16. Cu phthalocyanine -chitosan complex formation.

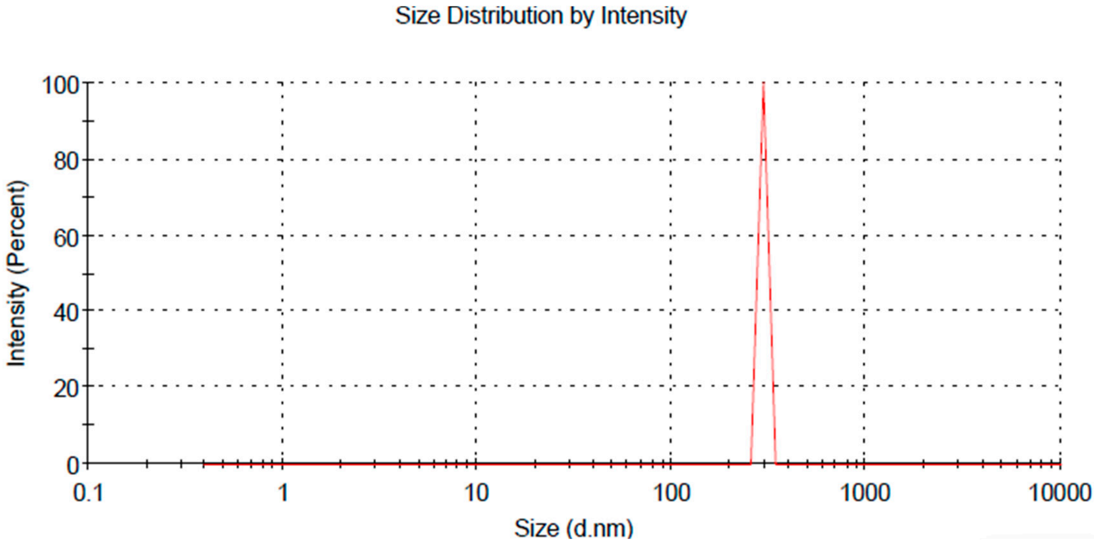

Figure S17. Mg phthalocyanine - chitosan complex formation.

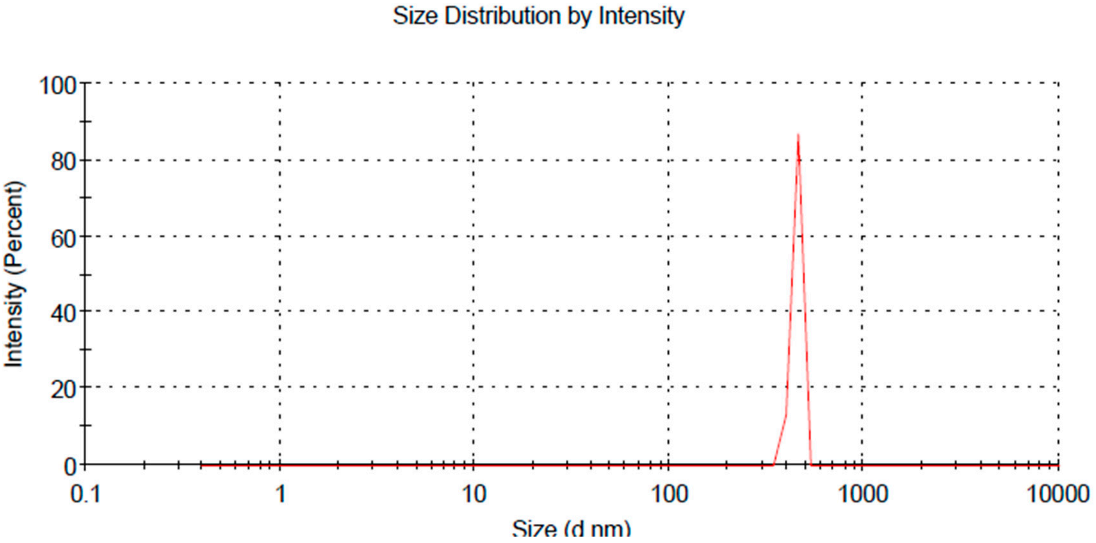

Figure S18. Ga phthalocyanine - chitosan complex formation.
